# Beneficial Bacteria and Plant Extracts Promote Honey Bee Health and Reduce *Nosema ceranae* Infection

**DOI:** 10.1007/s12602-022-10025-7

**Published:** 2023-01-13

**Authors:** Paula Melisa Garrido, Martín Pablo Porrini, Daniele Alberoni, Loredana Baffoni, Dara Scott, David Mifsud, Matín Javier Eguaras, Diana Di Gioia

**Affiliations:** 1 Instituto de Investigaciones en Producción Sanidad y Ambiente (IIPROSAM), CONICET, UNMdP, Centro Asoc. Simple CIC PBA, Funes 3350, Mar del Plata, Buenos Aires 7600 Argentina; 2 Centro de Investigación en Abejas Sociales (CIAS), FCEyN, UNMdP, Funes 3350, Mar del Plata, Buenos Aires 7600 Argentina; 3 Department of Agricultural and Food Sciences, University of Bologna, Viale Fanin 44, Bologna, 40127 Italy; 4 ADVANCE SCIENCE Ltd, Knocknacarra Rd, Galway, H91 XV84 Ireland; 5 Institute of Earth Systems, L-Università ta’ Malta, University Ring Rd, Msida, MSD2080 Malta

**Keywords:** Honey bees, Immune response, Nosemosis, *Nosema ceranae*, *Lactobacillus*, *Bifidobacterium*, *Lactiplantibacillus*, *Apilactobacillus*, Thymol, Seaweed, Lemongrass

## Abstract

The research aims to give new insights on the effect of administering selected bacterial strains, isolated from honey bee gut, and/or a commercial plant extract blend (HiveAlive^®^) on *Nosema ceranae*. Analyses were first performed under laboratory conditions such as different infective doses of *N. ceranae*, the effect of single strains and their mixture and the influence of pollen administration. Daily survival and feed consumption rate were recorded and pathogen development was analysed using qPCR and microscope counts. Biomarkers of immunity and physiological status were also evaluated for the different treatments tested using one bacterial strain, a mixture of all the bacteria and/or a plant extract blend as treatments. The results showed an increase of abaecin transcript levels in the midgut of the honey bees treated with the bacterial mixture and an increased expression of the protein vitellogenin in the haemolymph of honey bees treated with two separate bacterial strains (*Bifidobacterium coryneforme* and *Apilactobacillus kunkeei*). A significant effectiveness in reducing *N. ceranae* was shown by the bacterial mixture and the plant extract blend regardless of the composition of the diet. This bioactivity was seasonally linked. Quantitative PCR and microscope counts showed the reduction of *N. ceranae* under different experimental conditions. The antiparasitic efficacy of the treatments at field conditions was studied using a semi-field approach which was adapted from research on insecticides for the first time, to analyse antiparasitic activity against *N. ceranae*. The approach proved to be reliable and effective in validating data obtained in the laboratory. Both the mixture of beneficial bacteria and its association with Hive Alive^®^ are effective in controlling the natural infection of *N. ceranae* in honey bee colonies.

**Supplementary Information:**

The online version contains supplementary material available at 10.1007/s12602-022-10025-7.

## Introduction

Honey bee colonies are considered as superorganisms with a complex internal functional organisation, which have developed physiological mechanisms allowing colonies to avoid or cope with biotic and abiotic stresses [1]. This organisation implies complex population dynamics in case of diseases or parasitism. Nosemosis is a disease caused by the fungal microsporidians *Nosema apis* and *Nosema ceranae* (or *Vairimorpha apis* and *Vairimorpha ceranae* [2]). These microsporidia are intracellular obligate parasites that have exhibited negative synergistic effects with biotic and abiotic environmental conditions such as the presence of viruses [3], gut dysbiosis [4], pollen quality and diversity [5, 6], varroa [7], pesticides [8] and climate. The latter may play a major role, as it is capable of affecting the distribution, seasonality and severity of *N. ceranae* infections [9]. In fact, *N. ceranae* is adaptively advantaged to temperatures above the seasonal average [10]. The microsporidium strongly weakens colony health due to energetic stress in its hosts [11] and causes significant damage to the midgut [12] leading to a compromised nutrients intake and finally to honey bee starvation [13]. Increasing drought conditions in European regions [14] can negatively affect nectar flows [15] and in synergy with *N. ceranae*, negatively impacting honey bee populations. This synergy worsens the effects of nutritional stresses thus bringing the colony to a fast decline with consequent honey productivity loss [16] and may lead to queen bee replacement. *N. ceranae* also causes immune suppression during the first days of infection [17–19], advantaging other microbial pathogens or opportunistic parasites. The effects of *N. ceranae* on the humoral immune responses were also investigated (Antúnez et al. [17]). Several reports highlight the reduction of vitellogenin transcripts in infected honey bees [20–22]. The production of vitellogenin has multiple effects in honey bees [23], playing a key role in the regulation of division of labour, immunity and longevity [24, 25]. Currently, the most effective antibiotic able to control *N. ceranae* is the fumagillin dicyclohexylamine salt. Worldwide, beekeepers have relied on fumagillin treatments, even though toxic effects were reported on mammals upon short-term exposure [26, 27] and the treatment was observed to only be partially effective under determined conditions [28]. In relation to the apiculture sector in Europe, the 2001/82/EC Directive has effectively removed the antibiotic fumagillin from the European market; however, its use is still allowed in many countries. Considering that no alternatives seem to be available against *Nosema* sp., in early 2000s, several countries like UK, Spain, Belgium and Hungary had issued special authorisation to treat the disease in heavily infected apiaries with fumagillin [29]. On the other hand, a complex situation occurs on the American continent, insofar as many of the large honey-producing countries have prohibited, limited, or its use is still allowed even though the product is not commercially available. The need for alternative agents to control *Nosema* sp., preferably eco-friendly ones, is self-evident. A large number of plant extracts have been tested for their anti-microsporidian activity in *N. ceranae* infected honey bees, such as essential oils like thymol (from *Thymus vulgaris*) [30], laurel oil (from *Laurus nobilis*), oregano oil (from *Origanum vulgare*) and eucalyptus oil (from *Eucalyptus* spp.) [30] or plant ethanolic extracts [31] with contrasting results. Rice [32] reported that thymol significantly reduced *Nosema vespulae* spores load when tested under laboratory condition. Costa et al. [33] confirmed a significant reduction of *N. ceranae* spores after treating caged honey bees with thymol oil, also showing that *Nosema* inhibiting effect depends on the supply medium (e.g. sugar syrup works better than sugar candy). A 2-year investigation of the effects of HiveAlive^®^ induced a decrease in colony spore loads and significant increase in hive population over time, compared to control [34]. Another eco-friendly approach investigated in the last decade is the use of commensal and environmental bacteria supposed to be beneficial for honey bee health [35]. Interesting results have been obtained both by using bacterial metabolites and viable cells. A reduced *N. ceranae* spore number was observed by Porrini et al. [36] and Maggi et al. [37], following treatments of honey bees with *Bacillus subtilis* metabolites and lactic acid from *Lactobacillus johnsonii* CRL1647. Control of *N. ceranae* was observed by Audisio et al. [38] in hives treated with cell suspension of the same *L. johnsonii* strain. Baffoni et al. [39] obtained a reduction with both naturally and artificially infected bees with the use of a microbial formula containing Lactobacillaceae and *Bifidobacterium* spp. strains.

This work aims to explore an innovative approach to counteract *N. ceranae* proliferation in the honey bee gut, using an already developed bacterial mixture [39] and a plant extract blend possessing antifungal activity. The study considers different variables including natural and artificial *N. ceranae* infections, different concentrations of the infective inoculum, administration of single or multiple microbial strains, the bacterial mixture and the plant extract blend administered alone or combined together, as well as beebread absence in the diet. Laboratory studies were followed by in-field testing. The analysis of the efficacy of the proposed treatments, besides the definition of a strategy to counteract *N. ceranae*, will contribute to enlarge the knowledge on the parameters involved in the biological response of *Apis mellifera* to the administration of bacterial strains at laboratory and field levels.

## Methods

### Origin of the Tested Honey Bees

Experiments were carried out during 2018–2020 in CONICET (Consejo Nacional de Investigaciones Científicas y Técnicas) in the affiliate institution CIAS, Universidad Nacional de Mar del Plata, Argentina. Newly emerged hybrid honey bees (*Apis mellifera mellifera* x *Apis mellifera ligustica*) were obtained from brood frames of emerging honey bees picked from colonies located in the experimental apiary of J.J. Nágera Coastal Station ($38^\circ$ $10'06''$S, $57^\circ$ $38'10''W$). None of the selected colonies showed visible clinical symptoms of brood disease such as American Foulbrood and Chalkbrood or severe *Varroa destructor* presence [50].

### Preparation of the Bacterial Mixture, Plant Extract Blend and *Nosema ceranae* Inoculum

A selection of bifidobacteria (*Bifidobacterium asteroides* DSM 20431, *Bifidobacterium coryneforme* LMG 30569 and *Bifidobacterium indicum* DSM 20214) and lactobacilli (*Apilactobacillus kunkeei* LMG 30566, *Lactiplantibacillus plantarum* LMG 30567 and *Lactobacillus johnsonii* LMG 30568) was used in the experiment and grown on MRS in accordance with Olofsson et al. [40]. The plant extract product is a commercial product (Hive Alive^®^), marketed as an in-field treatment for improving honey bee health and controlling nosemosis disease over time when fed to honey bees in sugar syrup. The *N. ceranae* inoculum was obtained from infected bees. *N. ceranae* spores were multiplied in confined worker bees, collected, and purified and the specie was confirmed according to Martín-Hernández et al. [41]. The same *N. ceranae* spores stock was used for every infection.

### Toxicity and Palatability of Treatments

The toxicity and palatability of the plant extract blend on *A. mellifera* was systematically evaluated before the beginning of the experiments. The adequate concentration of the plant extract blend was selected. For that reason, honey bees were treated with 2.5 ml of Hive Alive^©^ per litre of sugar syrup (the recommended concentration by the manufacturer), 0.5 ml/l (1/5$\times$) and 12.5 ml/l (5$\times$). Mortality was recorded 9 days after the beginning of treatment.

### Honey Bees and Treatment Management in Cage Tests

Brood frames were kept in an incubator until honey bee emergence (32 ± 2 °C and 60% RH). Newly emerged honey bees were maintained at an average temperature of 29 ± 2 °C and 30% RH during every cage assay. Sugar syrup 1:1 (w/v) was used for the supplementation of feed additives or infection inoculum of *N. ceranae* spores. Worker bees were carefully removed from brood combs from the same colony and randomly confined and supplied with sugar syrup 1:1 (w/v) and water. Fifty individuals per treatment (three replicates) were confined to experimental cages. Confinement devices consisted in transparent and ventilated plastic jars (900 cm^3^) with inputs for gravity feeding devices and a removable side door [42]. Newly emerged honey bees were fed with freshly prepared spores of *N. ceranae* resuspended in sugar syrup according to Porrini et al. [43]. Each feeding treatment started at day 3 post-emergence, solutions were prepared in sugar syrup 1:1 (w/v), administered in gravity feeders. The content was replaced every day to estimate consumed amounts and evaporation in specific feeding devices. Two different incubators were used to avoid any influence of plant extract blend product vapours in the bacterial mixture test. Stored pollen (beebread) was used as a protein and lipids source in most of the experiments as pollen was found to deeply influence honeybees response to *N. ceranae *infection, affecting the disease severity [44]. It was manually collected from combs and slightly dried in an oven (50 °C for 24 h), homogenised and treated with UV radiation to diminish the viability of any possible *N. ceranae* spores present in the pollen [42]. In each assay, honey bees received potable tap water ad libitum. To estimate the basal infection in each assay, potentially due to food contamination, manipulation, or incidental ingestion of spores when the operculum is cut during bee emergence, DNA from newly emerged bees was analysed in qPCR (see “qPCR for Gene Expression Analysis’’ section).

### Experimental Design of Cage Tests

The experimental design is summarised in Table S1 which describes the variables considered in the different assays of the study.

#### Assay 1. Effect of Bacterial Mixture and Single Strains on Vitellogenin Expression

Newly emerged honey bees were placed into groups of 50 individuals per treatment per replicate. The following treatments were performed: a mixture of the six above-mentioned bacterial strains diluted in sugar syrup; the six individual bacterial strains each in separate dilute sugar solution; a control made up of sugar syrup mixed with the fermented MRS broth obtained from the bacterial mixture (FB) in equal volumes (1:1 v/v) and a negative control supplemented only with sugar syrup (C). A control that elicits an immune response was performed with the injection of sterile 0.15 M pH 7.5 phosphate-buffered saline solution (PBS) as described by Randolt et al. [45]. Single bacterial strains (without the fermented culture media) were diluted in a 2:1 (v/w) sugar syrup until an approximate concentration of 1 $\times$ 10^5^ CFU of single bacteria strains were obtained, in accordance with Evans and Lopez [46] whereas 6 $\times$ 10^5^ of the bacterial mixture was prepared and then resuspended in a sugar syrup solution. Water and UV-treated beebread were administered ad libitum. Five honey bees were sampled 4 days post-emergence, gut tissue was removed from abdomens and midguts were dissected and set aside for gene expression analysis. A pool of haemolymph from 10 individuals per replicate was collected and stored at −80 °C for further vitellogenin expression analysis by Sodium Dodecyl Sulphate - Polyacrylamide Gel Electrophoresis (SDS-PAGE).

#### Assay 2. Different Concentration of *N. ceranae* Spores

Honey bees were fed with sugar syrup and beebread in separate dispensing devices. After spore inoculation, feeding additives were administered ad libitum during the assay. A final mixture of bacterial mixture (1$\times$10^8^ CFU) was prepared and resuspended in a sugar syrup solution 1:1 (w/w). This assay took into consideration 15 experimental conditions with three replicates (Table S1): [C] control without infection; [N1] infection with 5 $\times$ 10^2^ *N. ceranae* spores; [N2] infection with 5 $\times$ 10^3^ *N. ceranae* spores; [N3] infection with 5 $\times$ 10^4^ *N. ceranae* spore. The ones involving feeding additives were given to infected honey bees: [B] bacteria mixture in combination [BN1; BN2 and BN3]; plant extract blend (2.5ml/l final concentration 0.082g/kg of sugar syrup) [HAN1; HAN2 and HAN3]; and the positive control [F] Fumagillin [FN1; FN2 and FN3]. Ten honey bees per experimental condition were sampled and guts were dissected, both at day 9 and day 14 after infection, for qPCR analysis in order to estimate *N. ceranae* units. A total of 360 honey bees were individually analysed in qPCR. Moreover, an additional 10 honey bees per experimental condition were sampled at the 14^th^ day for *N. ceranae* spores count with a haemocytometer under a light microscope as described by Cantwell [47].

#### Assay 3. Impact of a Beebread Diet on *N. ceranae* Development

and honey bee infection was performed as previously described. Fourteen treatments with three replicates were performed (Table S1). Experimental groups included the following: control without infection [C] and infection with 5 $\times$ 10^4^ *N. ceranae* spores [N]. Infected and non-infected bees were treated with the bacterial mixture [B] or plant extract blend (2.5ml/l, final concentration 0.082g/kg of sugar syrup) [HA]. As positive control for *N. ceranae* infection, fumagillin was used [F]. The MRS culture media of bacteria was used as further control. Combinations of *N. ceranae* infection with bacterial mixture, with plant extract blend or with fumagillin [BN; HAN; FN] including also a beebread diet [P] or not, were assessed. The midgut of 140 honey bees (10 per experimental condition) was dissected for qPCR analysis and 420 for microscope spore count on the ninth day post-infection.

#### Assay 4. Impact of Single Bacterial Strains vs Strain Mixtures on *N. ceranae* Development

Administration of additives consisted of the [C] control; [CN] *N. ceranae* infection with 5 $\times$ 10^4^ spores mixed with the [FN] fumagillin salt as well as the bacterial mixture and the single bacterial strains: [BN] bacterial mix (1 $\times$ 10^8^ CFU); [LKN] *Apilactobacillus kunkeei*; [LPN] *Lactiplantibacillus plantarum*; [LJN] *Lactobacillus johnsonii*; [BAN] *Bifidobacterium asteroides*; [BCN] *Bifidobacterium coryneforme*; [BIN] *Bifidobacterium indicum*. A final concentration of 5 $\times$ 10^7^ for each bacterial strains was prepared and resuspended for every strain except for *Bifidobacterium coryneforme* C155 which experienced growth problems and only a maximum of 2.1 $\times$ 10^6^ CFU could be reached. Ten honey bees for every experimental condition were sampled for qPCR analysis on the ninth day post-infection, for a total of 150 honey bees, individually analysed. Moreover, additional 10 bees per experimental condition and replicate were used for microscope spore count on individual midgut tissue, for a total of 450 honey bees.

### Assay 5. Impact of the Bacteria Mixture and the Plant Extract Blend on Honey Bees Artificially and Naturally Infected with *N. ceranae* in Field Conditions

Honey bees for the field assay were obtained from brood frames collected from the colonies of the experimental apiary (established 1 year prior to the start of the assays, including population and mated queens of the same genetic line). Newly emerged honey bees were incubated for 2 days at 30 ± 1 °C. Adult honey bees were divided in two groups of about 700 individuals each, half of which were artificially infected with *N. ceranae* spores (5 $\times$ 10^4^ spores/bee) [N]. Honey bees were marked with different colours on the thorax with a water based non-toxic paint (Enamel-Posca) before their introduction into the experimental colonies. Twelve experimental colonies in the apiary were selected. The experimental colonies hosting the painted bees were previously standardised for colony strength. Furthermore, colonies *N. ceranae* infection degree was estimated in accordance with Fries [48] as well as *Varroa destructor* infestation [49, 50]. Each colony hosted a group of 50 non-infected worker honey bees and a group of 50 artificially infected honey bees, identified by different colours as mentioned above. Selected colonies were further subdivided into four groups with the treatments sprayed directly on combs surfaces and honey bees. Each colony received the treatments (approximately 15 ml per comb), in four doses (once a week). Treatments consisted in sugar syrup: with no additives [C] and [CN]; with a mixture of bacteria [B] and [BN] at a final concentration of 2.3 $\times$ 10^6^ CFU of bacterial mixture; supplemented with the plant extract blend [HA] and [HAN] prepared according to the concentration used in the laboratory experiments (2.5ml/l, concentration 0.082 g/kg of sugar syrup) and with both the bacterial mixture and the plant extract blend [BHA] and [BHAN]. In this case the two additives were sprayed separately (12 h apart). Since [BHA] and [BHAN] were sprayed twice, with the aim of keeping the same conditions [C] and [CN]; [B] and [BN]; and [HA] and [HAN] also received the spraying of sugar syrup without feed additives on the same day. After releasing the two groups of marked honey bees (artificially infected and non-infected), honey bees were sampled twice, on the ninth and eighteenth days post-release, collecting 10 painted honey bees from each hive on each sampling day. The sampled honey bees, totalling 490, were individually analysed by qPCR for *N. ceranae* on the DNA extracted from their hindgut and rectum. Moreover, a maximum of 25 honey bees per hive/group were also collected for microscope spore count on midgut tissue on the eighteenth day.

### Molecular and Biochemical Methods

The full detailed molecular and biochemical methods are reported in Supplementary Material File.

#### qPCR for *N. ceranae* Unit Counts

DNA extraction of single honey bee guts was performed with a High Pure PCR template preparation kit (Roche Diagnostic). The 16S-like rRNA gene was selected to perform *N. ceranae* specific qPCR with primers designed by [28] (see Table S2). The reactions were carried out on StepOne thermal cycler (Applied Biosystems) relaying on SYBR Green chemistry. To determine the samples’ total *N. ceranae* a standard curve was prepared for absolute quantification. The melting curve was performed in each real-time reaction to assess amplicons melting temperature (73.77 ± 0.23 °C St. Dev) according to the genetic variability of *N. ceranae*. According to Cilia et al. [51], the variable number of 16S-like rRNA gene copies in *N. ceranae* ranges from 5.7 to 11.5 per genome [52]. Each sample output was divided by the average 16S-like rRNA gene copy number of 8.6 [52].

#### qPCR for Gene Expression Analysis

Total RNA was isolated from each individual bee using the RNeasy Mini Kit (Qiagen), following manufacturer’s instructions. RNA quantity was estimated using Qubit RNA BR Assay Kit (Thermo Fisher). The total RNA recovered was immediately used to generate cDNA using a commercial reverse transcription kit (Promega). Negative controls were run in parallel for each step (RNA extraction and reverse transcription reactions). The transcript levels for genes encoding the antimicrobial peptides abaecin, defensin, hymenoptaecin and vitellogenin were assessed using primers previously described [53, 54]. $\beta$-actin and RPS5 housekeeping genes were used as a reference gene [53, 55] (see Table S2). Real-time PCR reactions were carried out using QuantiTec SYBR PCR Kit (Qiagen). Reaction mixtures consisted in 1X QuantiTect SYBR Green PCR Master Mix, 0.5 ${\mu}$M of each primer (one pair of primers per reaction). RNase free water and 5 ${\mu}$l of 1:10 diluted cDNA in a final volume of 20 ${\mu}$l. PCR reactions were carried out using StepOne instrument (Applied Biosystems) and specificity of reactions was checked through melting curve analysis. The expression ratio between each target gene and the geometric mean of reference genes [56] was calculated according to the method described by Pfaffl [57].

### Relative Quantification of Vitellogenin

Honey bee haemolymph from assay 1 (“Assay 1. Effect of Bacterial Mixture and Single Strains on Vitellogenin Expression’’ section) was collected by puncturing the dorsal aorta using a glass microcapillary as described by Garrido et al. [58]. Haemolymph was pooled from 10 adults per replicate per treatment. The extraction process was carried out keeping samples in ice to prevent any degradation of the proteins. Twenty microliters of haemolymph were placed in 0.5 ml plastic tubes containing 79.5 ${\mu}$l of frozen buffer solution developed by Mead et al. [59] and adapted to our biological material. Details on SDS-PAGE are described in Supplementary Material.

### Statistical Analysis

In each experiment, mortality and food consumption was compared among the different groups by Log-Rank survival curves and Mann-Whitney tests respectively, unless otherwise noted. LC_50_ estimates and inverse 95% confidence limits (CL) for the plant extract blend were determined by Probit analysis, with adjustments for natural mortality. qPCR data were analysed with R software [60] and tested for normality and homoscedasticity with Shapiro and Levene’s tests. The dataset was corrected with Cooks Distance multivariate method, to identify outliers based on regression analysis comparison. Least Square Means for Multiple Comparisons statistical analysis was applied to all the datasets, together with a Bonferroni *p*-value correction for multiple comparisons. When necessary, statistical analysis was performed taking into consideration only similar groups with a longitudinal comparison (e.g. comparing treatments with bees infected with 500 *N. ceranae* spores and the control with the same infection dose). Relative vitellogenin expression was evaluated by one-way ANOVA. Comparisons between different treatments were performed by Dunnett’s multiple comparison test. *p*-values below 0.05 were considered significant. The variation in gene transcript levels among different groups was evaluated by the non-parametric Kruskal Wallis test. Comparisons between different treatments were performed by multiple comparisons of mean ranks and *p*-values were determined by using Bonferroni correction. The results were plotted using Prism software (GraphPad Software, version 6.0, San Diego, CA, USA).

## Results

### Effect of the Plant Extract Blend on Honey Bee Survival and Feed Consumption Rates in Non-infected Honey Bees

Following the administration of HA at different concentrations, low mortality was recorded at 24, 48 and 72 h. Therefore, LC_50_ could not be calculated by Probit analyses. When chronic oral toxicity was assessed during the 9 days of cage test, no differences between the survival curves were found at the different product concentrations when compared to the respective controls. Figures and statistical details on these preliminary assays are included in the supplementary material section (Fig. S1 and Table S1). Daily consumption rates did not differ among the different concentrations (Table S2). Based on these results, the intermediate dose of 2.5 ml/l was selected for further studies.

### Immune-Related Genes Expression and Levels of Vitellogenin in Haemolymph

Hymenoptaecin and vitellogenin relative expression in midgut did not significantly differ in any applied treatment (*p* $=$ 0.41 and *p* $=$ 0.70, respectively). However, buffer injection resulted in a significant increase in the expression of abaecin (*p* $=$ 0.01) and defensin (*p* $=$ 0.03) when compared to non-injected honey bees. Abaecin was also upregulated upon treatment with the bacterial mixture (*p* $=$ 0.04) in four-day-old honey bees (Fig. S2).

**Fig. 1:**
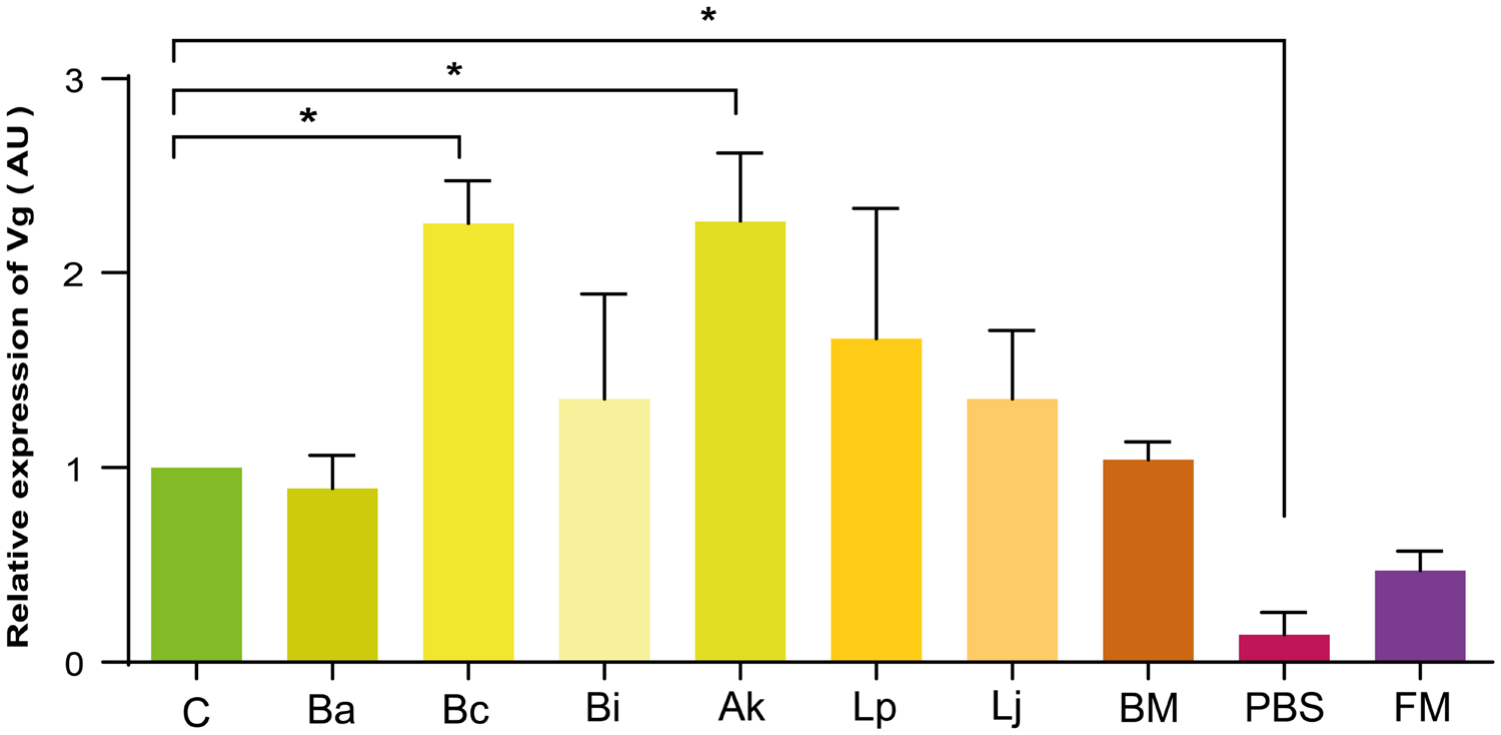
Relative expression of vitellogenin (Vg) in the hemolymph of adult honey bees, expressed in arbitrary units (AU). **[C]** sugar syrup diet, **[Bc]** Sugar syrup with *B. coryneforme* LMG 30569; **[Ba]** Sugar syrup with *B. asteroides * DSM 20431; **[Bi]** Sugar syrup with *B. indicum* DSM 20214;** [Ak] **Sugar syrup with *A. kunkeei* LMG 30566; **[Lp]** Sugar syrup with *L. plantarum* LMG 30567; **[Lj] **Sugar syrup and *L. johnsonii* LMG 30568; **[BM] **Sugar syrup with bacterial mixture; **[PBS] **sugar syrup diet and honey bees injected with PBS; **[FM]** Fermented media. There was placed 10 ${\mu}$g of total protein per lane. Asterisks indicate statistically significant differences when compared to the infected control (*p<0.05; ***p<0.01)

With regards to the relative quantification of the protein vitellogenin in haemolymph, a significant increase of its levels was observed in honey bees that received *B. coryneforme* and *A. kunkeei* (*p* $=$ 0.001 in both cases). Nevertheless, no changes were recorded with respect to the bacterial mixture (*p* $=$ 0.99) (Fig. 1). Interestingly, after 4 days from the aseptic injury with PBS, vitellogenin expression decreased significantly when compared to the control (*p* $=$ 0.03).

### Effects of the Additives on the Survival of Honey Bees Infected with *N. ceranae*

The infection with *N. ceranae* spores significantly reduced the longevity of honey bees when a diet based only on sugar syrup was administered (Fig. S3 and Table S3). However, when beebread was administered as complementary feed, the infection did not cause any significant effects on survival rates compared with non-infected bees, regardless of the infective doses. The combination of *N. ceranae* infection with BA, single bacterial strains and HA (assays 2–4) did not affect the survival rates in most cases. An exception was recorded in the experimental condition including BM plus beebread when compared to the control without beebread (Assay 3), in which an increase in mortality was observed. Survival curves and statistical details on these assays are included in the supplementary material section (Figs. S4–S6). Also, consumption (Tables S3, S7, and S10) and mortality rates (Table S6).

### Impact of Feed Additives on *N. ceranae* After Honey Bee Infection with Different Spore Doses

The basal infection of *N. ceranae*, measured by means of qPCR, resulted in Log 2.71 ± 0.27 *N. ceranae* units (NcU) with 4/10 positive honey bees per assay 2; Log 1.73 ± 0.70 NcU/honey bee with 4/10 positive honey bees per assay 3; Log 2.33 ± 0.33 NcU/honey bee with 6/10 positive honey bees for assay 4 and Log 3.95 ± 0.51 NcU for honey bees involved in the field assay, with 10/10 positive honey bees. *N. apis* was not detected in any of the analysed honey bees.

**Fig. 2:**
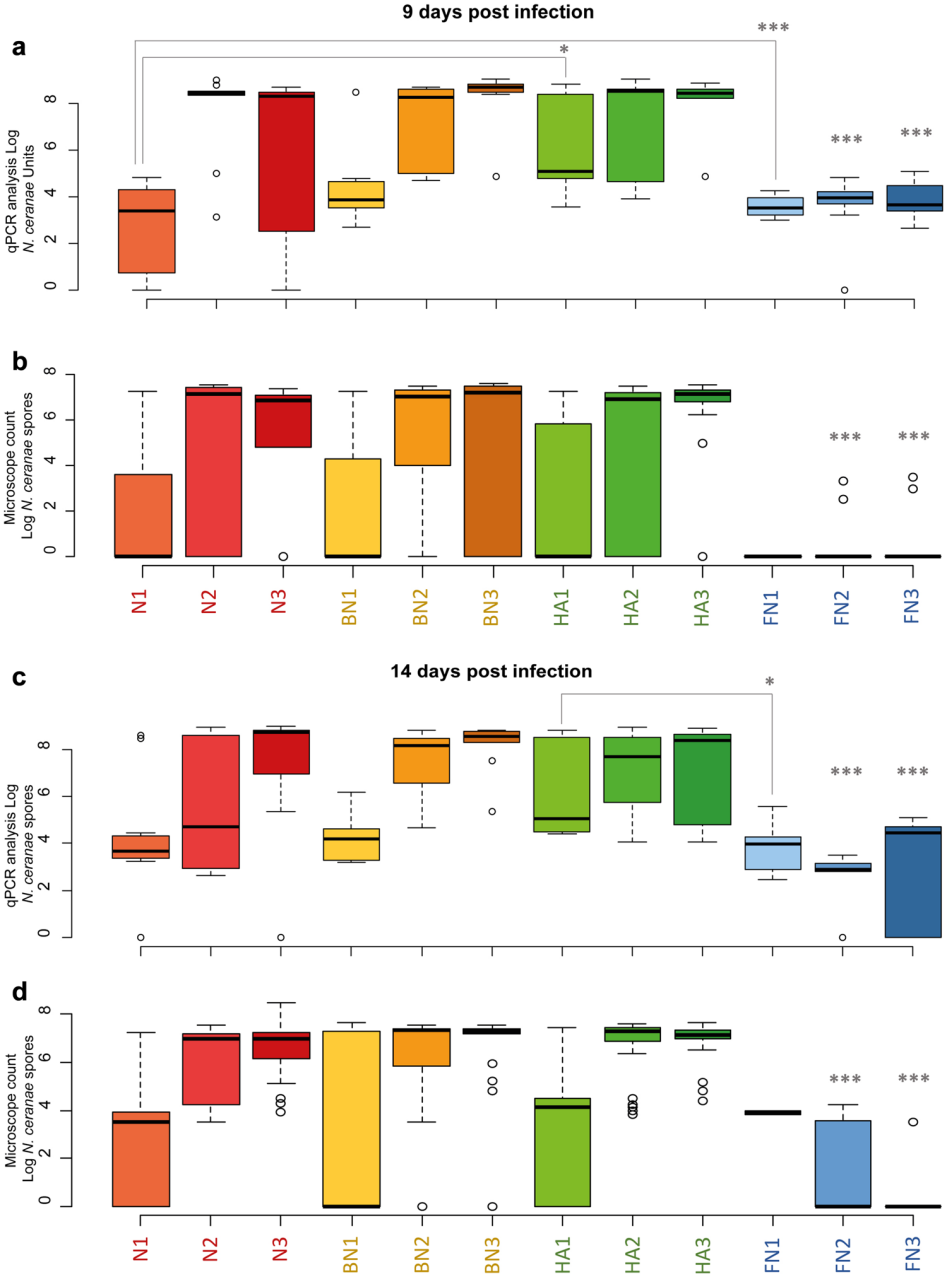
The boxplot chart shows the results of Assay 2 at two sampling times (T1 and T2). **a)** qPCR quantification of *N. ceranae *at T1; **b)** light microscope count of *N. ceranae* at T1; **c)** qPCR quantification of *N. ceranae* at T2; **d)** light microscope count of *N. ceranae* at T2. **[N1]** *N. ceranae* infection with 500 spores; **[N2]** *N. ceranae* infection with 5,000 spores; **[N3]** *N. ceranae* infection with 50,000 spores; **[B]** bacteria mixture $\times$ 500 spores **[BN1]**, $\times$ 5000 spores **[BN2]** or $\times$ 50,000 spores individually inoculated **[BN3]**; **[HA]** Hive Alive^®^ $\times$ 500 spores **[HAN1]**, $\times$ 5,000 spores **[HAN2]** or $\times$ 50,000 spores individually inoculated **[HAN3]**; **[F]** Fumagillin Salt $\times$ 500 spores**[FN1]**, $\times$ 5,000 spores**[FN2]** or $\times$ 50,000 spores individually inoculated **[FN3]**. Asterisks indicate statistically significant differences (*p<0.05; ***p<0.01). For treatments FN2 and FN3, *** indicate significant differences (***p<0.01) vs all treatments with the same invective dose of *N. ceranae*

The qPCR results on *N. ceranae* and the microscope spore counts related to assay 2 (Tables S4 and S5) are shown in Fig. 2a and 2c which report data on artificially infected honey bees at day 9 (T1) and day 14 (T2). At T1, samples obtained from treatments without infection (C, B and HA) showed similar *N. ceranae* values of Log 2.55 ± 0.45, 1.94 ± 0.20 and 1.69 ± 0.53 NcU/honey bee, respectively. Honey bees infected with different spore doses (N1, N2 and N3) showed a proportional increase in *N. ceranae* counts of Log 3.74 ± 1.04, 7.64 ± 1.95 and 8.05 ± 1.13 NcU/honey bee, respectively. The samples treated with the bacterial mixture (BN1, BN2 and BN3) also showed an increasing *N. ceranae* counts of Log 4.28 ± 1.60, 7.05 ± 1.88 and 8.32 ± 1.23 NcU/honey bee, respectively. The results for the treatments with the plant extract blend (HAN1, HAN2 and HAN3) showed *N. ceranae* counts of Log 5.89 ± 1.94, 7.27 ± 2.15 and 8.11 ± 1.15 NcU/honey bee, respectively. Unlike the other treatments, fumagillin (FN1, FN2 and FN3) showed similar values of parasite development regardless of the *N. ceranae* infective dose inoculated (Log 3.54 ± 0.41, 4.00 ± 0.43 and 3.79 ± 0.73 NcU/honey bee, respectively, Fig. 1a, c). The analysis of the whole dataset at T1 showed only two significant differences after the Bonferroni correction, both of them having the lower infection dose: HAN1 vs N1 (*p*<0.05), with an increase of NcU/bee upon treatment and FN1 vs N1 (*p*<0.01). Higher *N. ceranae* infection doses (5000 and 50,000) were significantly different in all experimental conditions when compared to fumagillin treatments (N2, BN2 and HAN2 vs FN2 (*p*<0.01); N3, BN3 and HAN3 vs FN3 (*p*<0.01).

At T2, treatments without infection (C, B and HA) showed *N. ceranae* counts values of Log 2.60 ± 1.20, 0.86 ± 0.84 and 1.50 ± 1.41 NcU/honey bee, respectively. N1 increased by 1 Log when compared with the first sampling (T1) reaching Log 4.75 ± 2.01 NcU/honey bee, although the difference was not significant. N2 dropped to Log 5.42 ± 2.59 NcU/honey bee at T2, but with high data dispersion. N3 increased from T1, reaching Log 8.40 ± 1.07 NcU/honey bee at T2. At T2, the *N. ceranae* load remained stable at Log 4.23 ± 0.95 NcU/honey bee in BN1, it slightly increased in BN2 whereas in BN3 it decreased with respect to T1; however, this was not statistically significant. Finally, FN1, FN2, and FN3 remained almost unvaried compared to T1. For every experimental condition standard, deviation and median are reported in the supplementary material section (Tables S4 and S5). The lower proliferation of *N. ceranae* in FN1 vs HAN1 (*p*<0.05) and all comparisons involving FN2 and FN3 vs all the respective experimental conditions with the same inoculation dose (N2, BN2, HAN2 and N3, BN3 and HAN3) were significant (*p*<0.01).

The results obtained from light microscopy analysis on individual midguts, expressed as exponential values of *N. ceranae* spores, are shown in Fig. 2b and 2d. As observed in qPCR results, the increase in the spore count between the infection with 5x10^3^ and 5x10^4^ spores was not always correlated with the increment of the inoculum (e.g. at day 9 the comparison between N2 vs N3 and at day 14 between HAN2 vs HAN3). The spore counts performed with data from day 9 and day 14 post-infection showed no effect of the potential antiparasitic treatments (HA and BM). On the other hand, spores in FN2 and FN3 were significantly reduced when compared to the respective doses of inoculum in N2, BN2, HAN2 and N3, BN3 and HAN3.

#### The Influence of Beebread When Combined with the Bacterial Mixture or the Plant Extract Blend on *N. ceranae* Infected Honey Bees

**Fig. 3:**
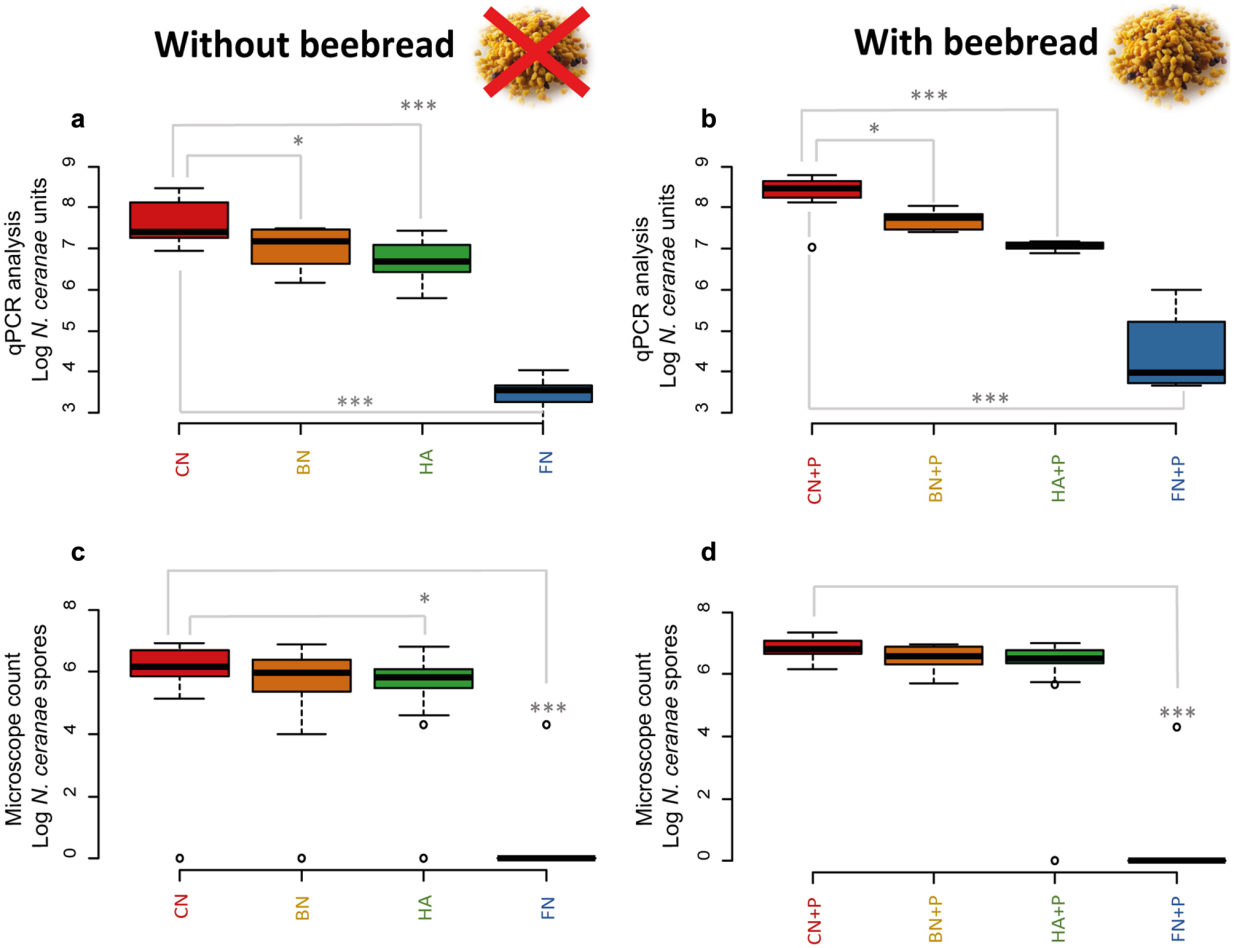
The boxplot chart shows the results of assay 3. **a)** and **c)** honey bees without beebread feed; **b)** and **d)** honey bees with beebread feed. Infected honey bees fed with only sugar syrup. All the honey bees were individually infected with 50,000 spores of *N. ceranae*** [N]**. **[CN]** no added treatment; **[HAN]** Hive Alive^©^; **[BN]** Bacterial mixture; **[FN]** Fumagillin Salt. Asterisks indicate statistically significant differences when compared to the infected control (*p<0.05; ***p<0.01)

In assay 3, infected honey bees that have received beebread showed a significant increase of the parasite in relation with infected honey bees with no beebread administration (Fig. 3a and b). The average increase was quantified in about 1 Log NcU/honey bee in qPCR (Table S8). The *N. ceranae* infected controls (CN and CN + beebread) showed a total abundance of 7.50 ± 0.54 and 8.33 ± 0.5 Log NcU/honey bee, respectively, which are the highest values recorded among the experimental groups. The experimental conditions comprising BN and BN + beebread showed a *N. ceranae* reduction to 7.01 ± 0.49 and 7.70 ± 0.22 Log NcU/honey bee, respectively, both significant (*p*<0.05) when compared to the infected controls. Also, HAN and HAN + beebread significantly decreased when compared to CN and CN + beebread, reaching values of 6.67 ± 0.51 and 7.06 ± 0.11 Log NcU/honey bee (*p*<0.01). Finally, spore counts in FN and FN + beebread strongly decreased (*p*<0.01) when compared to the respective controls. The increase in spores count upon beebread feeding was also observed with microscopy counts (Fig. 3c and d). Moreover, the statistical analysis on spore counts at day 9 showed significant results in HAN and FN vs CN (*p*<0.05 and *p*<0.01 respectively). The experimental conditions which included beebread showed a significant reduction only in FN + beebread vs CN + beebread (*p*<0.01, Table S9).

#### Effect of the Bacterial Mixture vs Single Bacterial Strains on *N. ceranae* Infection

**Fig. 4:**
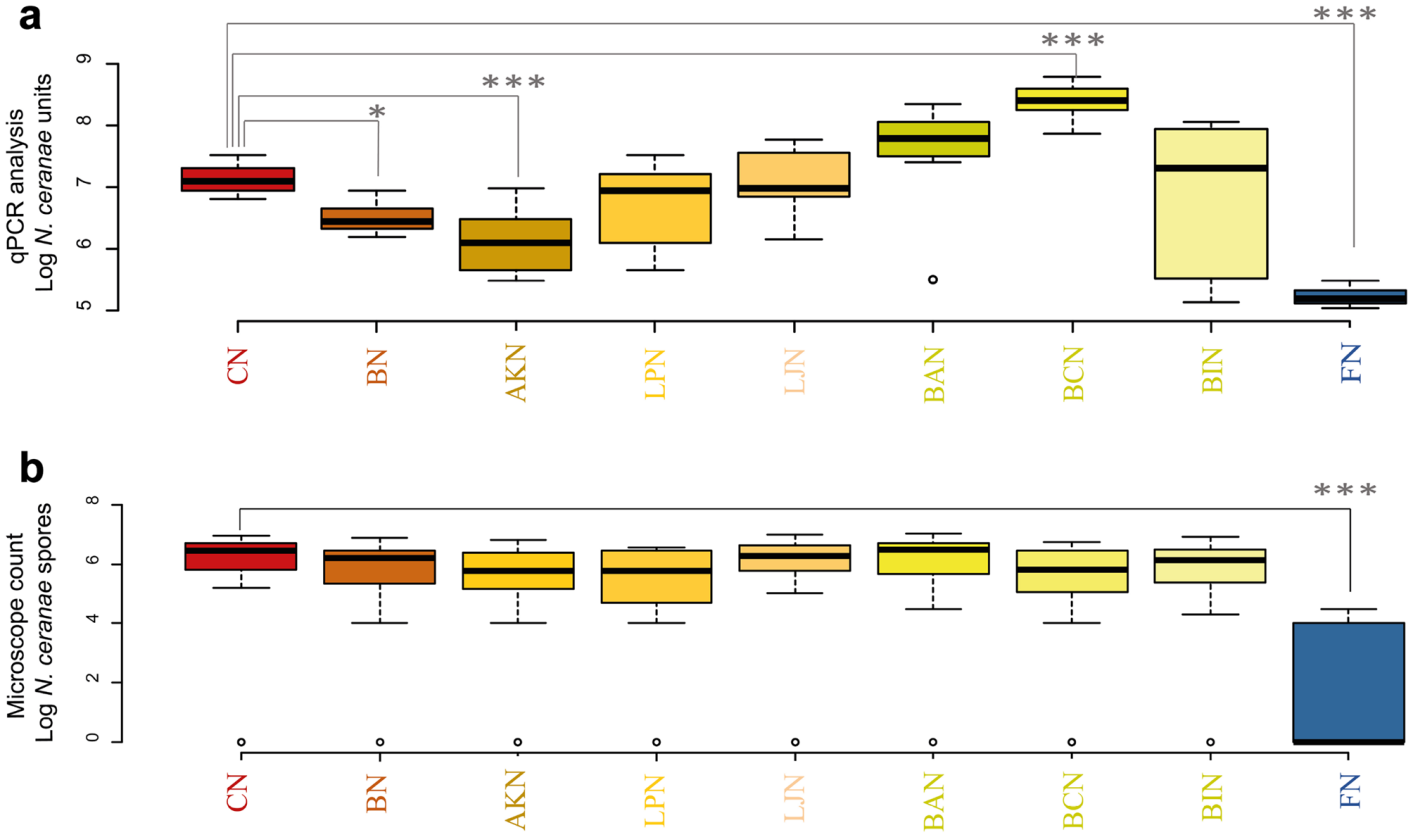
The boxplot chart shows the results of Assay 4. **a)** Results from qPCR analysis; **b)** Results from light microscope analysis. All the honey bees were individually infected with 50,000 spores of *N. ceranae* **[N]**. ** [CN]** Sugar syrup; **[BN]** Sugar syrup and bacteria mixture + 50,000 spores of*N. ceranae*; **[AKN]**: Sugar syrup and *A. kunkeei* LMG 30566 + 50,000 spores of *N. ceranae*;** [LPN]**: Sugar syrup and *L. plantarum* LMG 30567 + 50,000 spores of *N. ceranae*; **[LJN]**: Sugar syrup and *L. johnsonii* LMG 30568 + 50,000 spores of *N. ceranae*; **[BAN]**: Sugar syrup and *B. asteroides* DSM 20431 + 50,000 spores of *N. ceranae*; **[BCN]**: Sugar syrup and *B. coryneforme* LMG 30569 + 50,000 spores of *N. ceranae*; **[BIN]**: Sugar syrup and *B. indicum* DSM 20214 + 50,000 spores of *N. ceranae*. **[FN]**: Sugar syrup and antibiotic Fumagillin + 50,000 spores of *N. ceranae*. Asterisks indicate statistically significant differences when compared to the infected control (*p<0.05; ***p<0.01)

In assay 4, the qPCR analysis on *N. ceranae* units confirmed the effect of the BM against the *N. ceranae* infection, with *N. ceranae *in BN significantly lower when compared to CN (Fig. 4b, *p*<0.05) as already shown in assay 3. Regarding the effect of the single bacterial strains, the administration of *A. kunkeei* [AKN] resulted in a significant reduction of *N. ceranae* from 7.12 ± 0.24 to 6.13 ± 0.53 Log NcU/honey bee (*p*<0.01). Similarly, fumagillin [FN] significantly decreased the *N. ceranae* units (*p*<0.01). On the other hand, the administration of *B. coryneforme* [BCN] significantly increased the pathogen load to 8.38 ± 0.30 Log NcU/honey bee (*p*<0.01) while no significant increase was observed for the other microbial strains (BAN and BIN).

Microscope spore counts showed an increasing/decreasing trend similar to qPCR analysis (Fig. 4b). Statistical analysis showed significant differences only between fumagillin [FN] and the control [CN] (*p*<0.01, Tables S11 and S12).

#### Effect of Bacteria and/or a Plant Extract Blend on *N. ceranae* Development In-field Test

The experiment was performed by means of releasing marked bees (Table S1), following a semi-field experimental approach. The average *N. ceranae* spores load in forager honey bees collected from each selected experimental colonies was 1.8 $\times$ 10^6^ ± 0.3 $\times$ 10^6^. Moreover, the Varroa mite infestation percentage was 0.1 ± 0.05%.

The recovery of marked bees was carried out successfully on the ninth day post-release for all the experimental conditions, whereas difficulties were encountered in sampling bees on the eighteenth day for the BHAN and HAN conditions. Although honey bees under every condition tested developed nosemosis during the experiment, the highest amount of the parasite was recorded for the group of artificially infected honey bees.

**Fig. 5:**
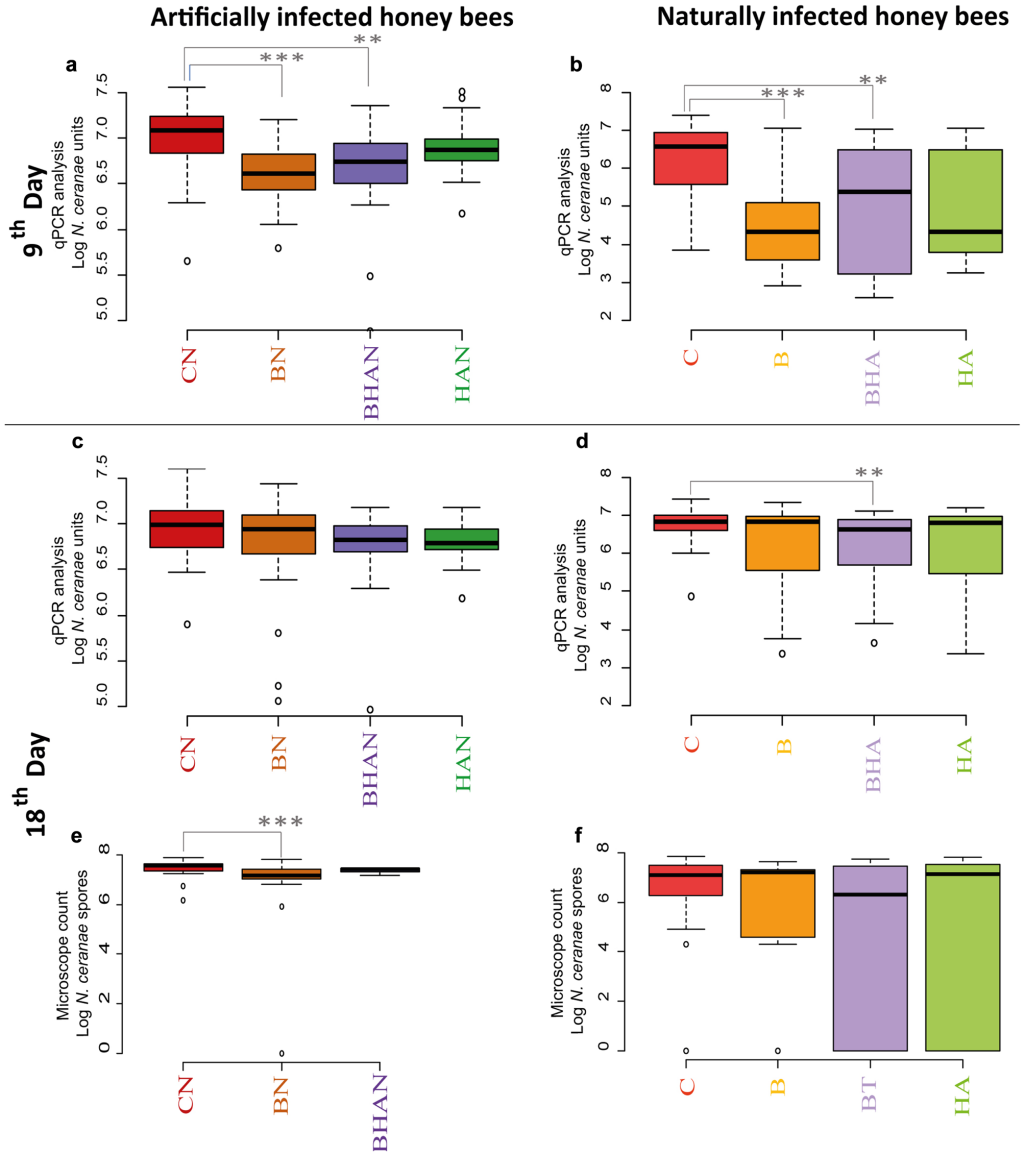
The boxplot chart shows the results of assay 5, under field conditions. Measurements on parasite development were performed by means of qPCR (figures **a**, **b**, **c**, and **d**) or light microscope (figures **e** and **f**).** a)** Artificially infected honey bees sampled at the ninth post-infection; **b)** Naturally infected honey bees sampled at the ninth post-release in the colony; **c)** and **e)**. Artificially infected honey bees sampled on the eighteenth day post-infection;** d)** and **f)**, Naturally infected honey bees sampled on the eighteenth day post-release in the colony. **[N]** *N. ceranae* infection of 50,000 spores individually inoculated before release; The sprayed treatments consisted in **[C]** Sugar syrup; **[B]** Sugar syrup and bacteria mixture; **[HA]** Sugar syrup and the plant extract blend; and **[BHA]** Sugar syrup, bacteria mixture and the plant extract blend. Asterisks indicate statistically significant differences when compared to the infected control (*p<0.05; ***p<0.01)

The qPCR analysis performed on the honey bees recovered on the ninth day post-infection indicated high effectiveness against *N. ceranae* development of BM and the combination of the mixture with HA in artificially (BN and BHAN, *p*<0.01 and *p*<0.05, respectively) and naturally infected bees (B and BHA, *p*<0.01 and *p*<0.05, respectively) in comparison to their respective controls (Fig. 5a, b). The qPCR analysis performed on the honey bees recovered on the eighteenth day showed a significant reduction in NcU (Fig. 5d), only for the treatment combining HA with bacteria (BHA) in naturally infected honey bees, when compared to the control (*p*<0.05). Tables S13 and S14 in the supplementary material section include details on average values and statistical analysis. The results of the microscope counts on the midgut of honey bees recovered on the eighteenth day showed a significant reduction on *N. ceranae* spore counts for the artificially infected bees treated with bacteria (BN) (*p*<0.01). At this sampling time, a minimum of 10 worker bees were recovered for each condition (Table S13). The low quantity of marked worker bees for the HAN experimental condition did not allow the performance of statistical analysis.

## Discussion

In the last decade, many studies have approached the application of probiotics and plant extracts to improve honey bee health and mitigate diseases affecting colonies [33, 38, 46, 61–65]. The present study not only provides evidence supporting the effect of the mixture of Lactobacillaceae and *Bifidobacterium* strains (BM) and a commercial plant extract blend (HA) against nosemosis, but also defines an experimental approach to assess some of the main variables influencing the additive bioactivity on parasite development. In previous studies, survival tests based on feeding single bacterial strains or BM to honey bees under laboratory conditions showed differing results, spanning from the enhancement of survival to toxicity, depending on the administered strains and their concentrations [39, 62, 66, 67]. In this work, the survival rates obtained after a mid-term administration of bacteria and HA were promising, showing that both, combined or not with beebread, did not cause significant lethal or adverse effects on adult honey bees and were consumed. We also tested whether the composition of BM or each single bacterial strain has sustained effects on bee physiology such as the levels of vitellogenin (Vg), a marker for the overall honey bee health and the expression of antimicrobial peptides (AMPs), which are crucial components of insect innate immunity [20]. The results show that single strains did not alter significantly the AMPs transcripts, in agreement with the results obtained by Arredondo et al. [62] with different *A. kunkeei* strains. However, abaecin was upregulated in honey bees upon the ingestion of BM. A similar result, but on honey bee larvae, was also reported by Evans and Lopez [46]. In this work, no significant changes of vg transcripts in the midgut were shown among the different experimental conditions. Besides, analysis of circulating Vg in haemolymph was considered essential and allowed the conclusion that the BM did not alter the expression of this protein, in agreement with Tlak Gajger et al. [67]. Nevertheless, honey bees that received *A. kunkeei* or *B. coryneforme* in their diets significantly expressed more Vg in the haemolymph although it remains unclear whether these bacterial strains are involved in the modulation of this protein, which, in turn, governs a variety of physiological aspects [24, 25]. When performing artificial infection, one critical aspect was the selection of the initial infective dose of *N. ceranae*. Wide ranges of *Nosema* sp. spores, between 10,000 to 1,000,000 spores/honey bee have been used for the infection, as reviewed by Martín-Hernández et al. [68], with results that vary considerably. In the present work, the results show that the initial dose of 500 spores was able to begin an infection in every individual analysed, although detectable only with qPCR and not always by means of light microscopy. In the first experiment, regardless of the amount of the inoculum administered, the application of both the BM and HA did not counteract the development of *N. ceranae*. However, Baffoni et al. [39] and Charistos et al. [34] reported a completely opposite outcome for cage tests based on BM and for field tests with HA respectively. These differences could be ascribed to the seasonality since winter honey bees, as those used in this test, show different physiological features with respect to summer ones [69] and respond differently to feed treatments as already observed in other studies [70, 71]. Based on the consideration mentioned, it was decided to standardise further experiments to 50,000 spores and to only use summer worker bees. In the following assay, the use of the feed additives was effective in diminishing *N. ceranae* units in all the experimental conditions. Microscope spore counts, however, were significantly reduced only under HAN and FN vs CN, showing differences in the performance of the two applied quantitative techniques [72]. Moreover, HA proved to be more effective against *N. ceranae* than BM, with significant results obtained when analysing samples with both quantitative methods. This result complements the information reported by the HiveAlive^®^ manufacturer about the best efficacy obtained when used consistently over time in the field. Besides, it was confirmed that beebread increased the proliferation of *N. ceranae* without compromising honey bee longevity, a phenomenon already reported in the literature [5, 73, 74]. The results of testing the effect on parasite development of single strains of the BM showed that *A. kunkeei* was even more effective than the multi-strain mixture in reducing the *N. ceranae* load. However, *B. coryneforme* increased *N. ceranae* units, maybe buffering the effect of BM. In line with this, several *A. kunkeei* strains were shown to exert a prophylactic action against fungal pathogens restoring the symbiotic communities of the gut in case of dysbiosis [75] and decreasing the counts of *N. ceranae* spores [62].

Although cage tests are very useful, impair important limitations on social insects like honey bees [76, 77]. The field tests confirmed some of the results obtained under laboratory conditions such as the *N. ceranae* reduction in naturally and artificially infected honey bees that received the BM alone or in combination with HA. Previous trials using HA showed an efficacy against *N. ceranae* when the product was supplied by feeding, according to manufacturer instructions [34]. Here, the concentration used was four times lower than the maximum concentration recommended for administrations by spraying, which could have influenced the final efficacy reached. The selection was done according to the feeding concentration employed in the laboratory and to avoid possible unwanted interactions in combination with bacteria treatment. However, showed a significant *N. ceranae* reduction after 18 days when naturally infected worker bees were analysed, showing a possible joint action of both treatments. On the other hand, the expected quantity of marked individuals at the first sampling time was recovered, whereas at the second one, less honey bees could be recovered from the artificially infected group of worker bees, which is concordant with the reported effect that *Nosema* sp. infection impairs on survival and homing capacity of honeybees [78–81]. This loss of individuals mainly was observed on strongly infected honey bees treated with HA. In-field experiments showed positive results on the colonies’ population parameters after administering a bacterial strain for three or more months with inner feeders [38, 82]. However, the promising results obtained in our assay after only 3 weeks of treatment, coupled with the administration mode (spraying in situ), demonstrate a less time-consuming approach that deserves experimentation on a larger scale.

The present research has given new insights on aspects related to *N. ceranae* infections which have been scarcely or never even considered in studies testing the antiparasitic efficacy of substances, such as the impact of the amount of *N. ceranae* spores in artificially infected honey bees, the effect of beebread when combined anti-*N. ceranae* treatments and the potential of single strains vs a BM in counteracting nosemosis. Furthermore, laboratory and in-field test indicated a promising efficacy of HA and BM against the microsporidium proliferation, since they both resulted in a significant reduction of the parasite in the short term. Therefore, our study has contributed to the understanding that the bacterial strains selection, as well as the time and frequency of the administration strongly influence the results of the treatments. It is evident that disease control in bee colonies under a productive management environment involves not only the antiparasitic and biological effect of the substances administered, but also the complex interaction between the honey bee and its parasites.

### Supplementary Information

Below is the link to the electronic supplementary material.

Supplementary file1 (PDF 755 KB)

## Data Availability

Raw data not published in supplementary materials are available on reasonable request from the corresponding author.
