# Supplementary material for: Beneficial Bacteria and Plant Extracts Promote Honey Bee Health and Reduce Nosema ceranae Infection
Source: Probiotics Antimicrob Proteins. 2023 Jan 13;16(1):259–74. doi: 10.1007/s12602-022-10025-7 (PMC10850026; doi:10.1007/s12602-022-10025-7)
Supplement: Supplementary file 1 — Supplementary file1 (PDF 755 KB) [file 12602_2022_10025_MOESM1_ESM.pdf]

Supplementary Material of:

Beneficial bacteria and plant extracts promote honey bee health and reduce *Nosema ceranae* infection

Paula Melisa Garrido[1,2], Martín Pablo Porrini[1,2], Daniele Alberoni[3*], Loredana Baffoni[3], Dara Scott[4], David Mifsud[5], Martín J. Eguaras[1,2], Diana Di Gioia[3]

[1]Instituto de Investigaciones en Producción Sanidad y Ambiente (IIPROSAM), CONICET, UNMdP, Centro Asoc. Simple CIC PBA, Funes 3350, Mar del Plata; Buenos Aires, 7600, Argentina.

[2]Centro de Investigación en Abejas Sociales (CIAS), FCEyN, UNMdP, Funes 3350, Mar del Plata, Buenos Aires, 7600, Argentina.

[3]Department of Agricultural and Food Sciences, University of Bologna, Viale Fanin 44, Bologna, 40127, Italy.

[4]ADVANCE SCIENCE Ltd, Knocknacarra Rd, Galway, H91 XV84, Ireland.

[5]Institute of Earth Systems, L-Università ta' Malta, University Ring Rd, Msida, MSD2080, Malta

***Corresponding author:**
Daniele Alberoni
daniele.alberoni@unibo.it
+39 051 2096269

Supplementary data on experimental procedures

1. Cage test experimental design

Table S1. Summary of the cage test experimental design

Assay 1	Sugar Syrup	Bacterial Mixture	Single bacterial strain	Phosphate Saline Buffer	Fermented broth
C	✓				
PBS	✓			✓	
B	✓	✓			
LK	✓		✓		
LP	✓		✓		
LJ	✓		✓		
BA	✓		✓		
BC	✓		✓		
BI	✓		✓		
FB	✓				✓

Assay 2	Sugar Syrup	Pollen	*N. cernane* 500	*N. cernane* 5,000	*N. cernane* 50,000	Bacterial mixture	Hive Alive®	Fumagilin
C	✓	✓						
B	✓	✓				✓		
HA	✓	✓					✓	
CN1	✓	✓	✓					
CN2	✓	✓		✓				
CN3	✓	✓			✓			
BN1	✓	✓	✓			✓		
BN2	✓	✓		✓		✓		
BN3	✓	✓			✓	✓		
HAN1	✓	✓	✓				✓	
HAN2	✓	✓		✓			✓	
HAN3	✓	✓			✓		✓	
FN1	✓	✓	✓					✓
FN2	✓	✓		✓				✓
FN3	✓	✓			✓			✓

Assay 3	Sugar Syrup	Pollen	Media MRS	*N. cernane* 50,000	Bacterial mixture	Hive Alive®	Fumagillin
C	✓		✓				
CP	✓	✓	✓				
B	✓		✓		✓		
BN	✓		✓	✓	✓		
B+P	✓	✓	✓		✓		
BN+P	✓	✓	✓	✓	✓		
CN	✓			✓			
CN+P	✓	✓		✓			
HA	✓					✓	
HAN	✓			✓		✓	
HA+P	✓	✓				✓	
HAN+P	✓	✓		✓		✓	
FN	✓			✓			✓
FN+P	✓	✓		✓			✓

Assay 4	Sugar Syrup	*N. cernane* 50,000	Bacterial mixture	Single bacterial strain	Fumagilin
C	✓				
CN	✓	✓			
BN	✓	✓	✓		
LKN	✓	✓		✓	
LPN	✓	✓		✓	
LJN	✓	✓		✓	
BAN	✓	✓		✓	
BCN	✓	✓		✓	
BIN	✓	✓		✓	
FN	✓	✓			✓

Assay 5	Sugar Syrup	*N. cernane* 50,000	Bacterial Mixture	Hive Alive
C	✓			
CN	✓	✓		
B	✓		✓	
BN	✓	✓	✓	
HA	✓			✓
HAN	✓	✓		✓
BHA	✓		✓	✓
BHAN	✓	✓	✓	✓

2. Supplementary data on DNA extraction and qPCR procedures

DNA extraction of single honey bee guts was performed with a High Pure PCR template preparation kit (Cas. 11796828001; Roche Diagnostic; Buenos Aires, Argentina) following manufacturer instructions with little modifications. The midgut and rectum were manually macerated with plastic micro pestles in 200µL buffer solutions. The 16S-like rRNA gene was selected to perform *N. ceranae* specific qPCR relaying on specific primer Nc841f 5'-GAGAGAACGGTTTTTTGTTTGAGA-3' and Nc980r 5'-ATCCTTTCCTTCCTACACTGATTG-3' [28]. The reactions were carried out on StepOne thermal cycler (Applied Biosystems) with a standard two-step PCR method using SYBR Green PCR Master Mix (Life Technologies, Milan, Italy). The annealing temperature was set at 61ºC for 25 seconds, 45 cycles. To determine the samples' total *N. ceranae* 16S-like rRNA gene copy number, a standard curve was prepared as follows: the target gene was PCR amplified with HI-FI Polymerase (PCR-BIO, London, United Kingdom), the reaction product verified on agarose gel for its bp length (146 bp), then purified with the PCR clean up kit (Macherey-Nagel, Duren, Germany) and finally quantified with Qubit v.3. Randomly selected samples were analysed in order to detect a co-infection with *N. Apis* using specific primers [28].
The standard PCR fragment was diluted to 1:10 to obtain the reference standards. The melting curve was performed in each real-time reaction to assess amplicons melting temperature (73.77 ± 0.23 ºC St. Dev) according to the genetic variability of *N. ceranae*. According to Cilia et al. [51], the variable number of 16S-like rRNA gene copies in *N. ceranae* ranges from 5.7 to 11.5 per genome [52]. Each sample output was divided by the average 16S-like rRNA gene copy number of 8.6 [52].

To perform RNA isolation and cDNA synthesis, 600 µL of RLT buffer (Qiagen) were added to samples. These were disrupted and they were disrupted and homogenised using a sterile plastic micro-pestle and a sterile plastic tube. Total RNA was isolated from each individual bee using the RNeasy Mini Kit (Cas. 74104, Qiagen), following manufacturer's instructions. A DNase digestion treatment was inserted in the protocol, adding 10 µL of DNase and 70 µL of buffer RDD per sample (Quiagen). RNA quantity was estimated using Qubit RNA BR Assay Kit (Cas.Q10211, Thermo Fisher). The total RNA

recovered was immediately used to generate cDNA using a commercial reverse transcription kit (Cas. A3802, Promega), used according to manufacturer's instructions. Obtained cDNA was stored at -20ºC $. Negative controls were run in parallel for each step (RNA extraction and reverse transcription reactions). In order to evaluate the effect of the administration of midgut bacterial symbionts on the immune response of honey bees, the transcript levels for genes encoding the antimicrobial peptides abaecin, defensin, hymenoptaecin and vitellogenin were assessed using primers previously described [53, 54]. β-actin and RPS5 housekeeping genes were used as a reference gene [55] (Table S2). Real-time PCR reactions were carried out using QuantiTec SYBR PCR Kit (Qiagen), according to manufacturer's recommendations. Reaction mixtures consisted in 1X QuantiTect SYBR Green PCR Master Mix, 0.5 $\upmu$M of each primer (one pair of primers per reaction). RNase free water and 5 µL of 1:10 diluted cDNA in a final volume of 20 µL. PCR reactions were carried out using StepOne instrument (Applied Biosystems) and the cycling programme consisted in an initial activation step at 95ºC for 10 min, and 45 cycles of 95ºC for 15 s, 59ºC for 25 s and 95ºC for 15 s. Specificity of reactions was checked through melting curve analysis. The expression ratio between each target gene and the geometric mean of reference [56] was calculated according to the method described by [57].

Table S2. List of primers used in this experiment to carry out quantification of specific microsporidia targets, and detection for the gene expression analysis.

	Primer Name	Sequence (5′-3′)	Amplicon size (bp)	Reference
**β-Actina**	βAct_f	TTGTATGCCAACACTGTCCTTT	120	[55]
	βAct_r	TGGCGCGATGATCTTAATTT		
**Ribosomal Protein S5**	RPS5_f	AATTATTTGGTCGCTGGAATTG	115	[56]
	RPS5_r	TAACGTCCAGCAGAATGTGGTA		
**Abaecin**	ABA_f	CAGCATTCGCATACGTACCA	72	
	ABA_r	GACCAGGAAACGTTGGAAAC		
**Defensin-1**	Def1_f	TGCGCTGCTAACTGTCTCAG	119	
	Def1_r	AATGGCACTTAACCGAAACG		
**Hymenoptericin**	Hym_f	CTCTTCTGTGCCGTTGCATA	200	
	Hym_r	GCGTCTCCTGTCATTCCATT		
**Vitellogenin**	Vit_f	GCAGAATACATGGACGGTGT	146	[54]
	Vit_r	GAACAGTCTTCGGAAGCTTG		
Nosema apis	Na65_f	CGTACTATGTACTGAAAGATGGACTGC	116	[83]
	Na181_r	AGGTCTCACTCTTACTGTACATATGTTAGC		
Nosema ceranae	Nc841_f	GAGAGAACGGTTTTTTGTTTGAGA	140	
	Nc980_r	ATCCTTTCCTTCCTACACTGATTG		

3. Supplementary data on relative quantification of vitellogenin

Honey bee haemolymph from assay 1 (Section 2.5.1) was collected by puncturing the dorsal aorta using a glass microcapillary as described by Garrido [58]. Haemolymph was pooled from 10 adults per replicate per treatment. The extraction process was carried out keeping samples in ice to prevent any degradation of the proteins. Twenty microliters of haemolymph were placed in 0.5 ml plastic tubes containing 79.5 µL of frozen buffer solution developed by Mead [59] and adapted to our biological material (to prevent samples melanisation) together with 1mM of protease inhibitor (phenylmethylsulfonyl fluoride). The samples were centrifuged at 8000 rpm at 4ºC $ for five minutes to remove haemocytes. An aliquot of supernatant was used to determine the total protein concentration by the Bradford method and was performed in triplicate at 595 nm using SimplyBlue SafeStain (Invitrogen Cas. LC6060). A standard curve was built with bovine serum albumin as a protein standard. To store homogenates for protein analysis, LDS Sample Buffer 5× was added (Bolt, Invitrogen) then samples were boiled for two minutes and frozen at -20ºC $. Samples denatured in a boiling water bath with 1× LDS Sample Buffer for five minutes and equal quantities of protein (5 µg of total protein/well) were loaded on 4-12% Bis-Tris Plus gels (Bolt, Invitrogen) by using Mini Gel Tank apparatus (Invitrogen). SDS-PAGE was carried out at 170 V for one hour in 1× running buffer. Pre-stained molecular mass standards, ranging from three to 198 KDa (SeeBlue Plus2, Novex, Life Technologies) were run in each gel. Honey bee relative protein levels were determined by densitometry with the software ImageJ.

4. Supplementary data on quantification of *N. ceranae* development, consumption of the treatments and toxicity on honey bees for every assay.

4.1. Results and statistical details on "Preliminary toxicity tests"

4.1.1. Survival

Figure S1

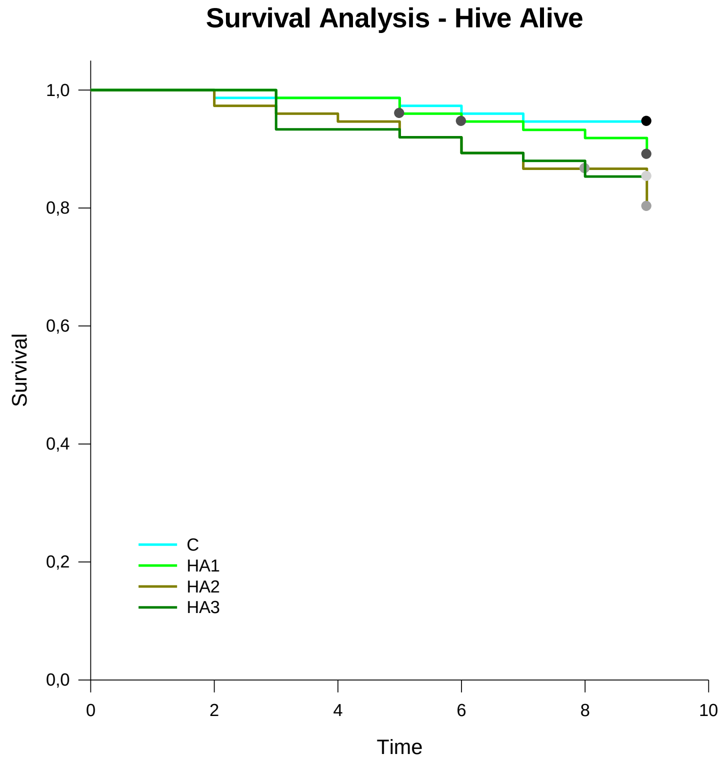

Figure S1. Chronic toxicity of the commercial product based on plant extracts (HA). Honey bees were treated control (C) sugar syrup with 0.5 ml of Hive Alive® per liter of sugar syrup (HA1), 2.5 ml/l (HA2) equal to the concentration recommended by manufacturer and 12.5 ml/l (HA3). Mortality was recorded during 9 days after the beginning of the treatment. No differences between the survival curves were found (Log-Rank Test, Statistic= 6.922, DF: 3, p-value= 0.074).

Table S3

	**Survival Time**	**Std. Error**	**95% Conf. Lower**	**95% Conf. Upper**	**Stat. dif. Vs. control (C)?**
**C (P)**	8.787	0.133	8.526	9.047	-
**HA1 (P+T ×1/5, 0.5 ml/l)**	8.731	0.125	8.485	8.977	no
**HA2 (P+T ×1, 2.5 ml/l)**	8.427	0.192	8.051	8.803	no
**HA3 (P+T ×5, 12.5 ml/l)**	8.413	0.195	8.031	8.795	no

Table S3. Statistical details for preliminary toxicity tests. Comparison for survival curves (Log-Rank Test, Statistic= 6.922, DF: 3, p-value= 0.074). Pairwise multiple comparison was performed with Holm-Sidak method. Overall significance level of 0.05. Abbreviations, **P:** pollen; **HA:** Hive Alive®.

4.1.2. Consumption rate

Table S4

	C (P)	HA1 (P+T ×1/5, 0.5 ml/l)	HA2 (P+T ×1, 2.5 ml/l)	HA3 (P+T ×5, 12.5 ml/l)
**Avg.**	33.12	36.15	35.52	30.17
**Dev.**	4.34	4.98	3.39	2.47

Table S4. Average of consumption rates for each treatment (mg/bee/day). No statistical differences were found (ANOVA, Statistic: 1.123, DF: 5, p value: 0.39). Overall significance level of 0.05. Abbreviations, **P:** pollen; **HA:** Hive Alive®.

4.2. Results and statistical details on “Assay 1”

Figure S2

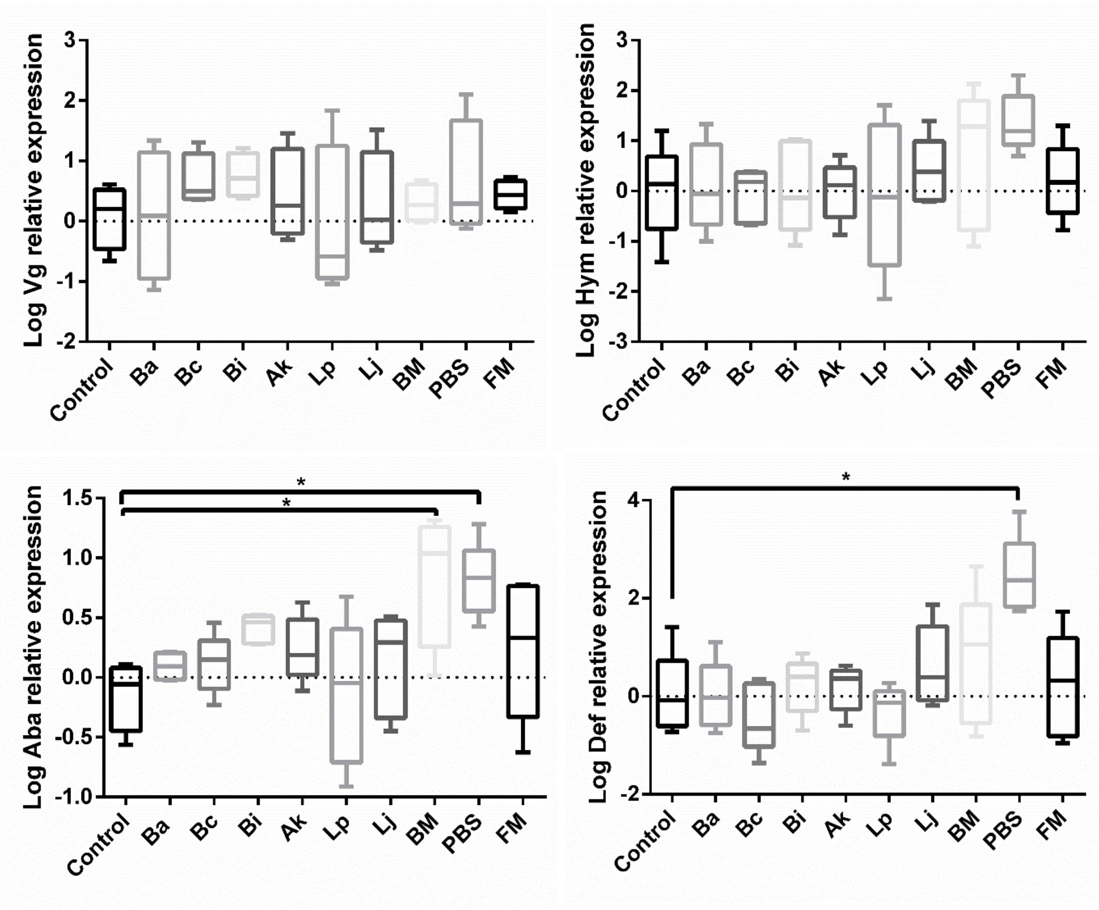

Figure S2. Relative mRNA levels of AMPs and vitellogenin genes in the midgut of *A. mellifera* by different treatments. **[C]** sugar syrup diet; **[Bc]** Sugar syrup with *B. coryneforme* LMG 30569; **[Ba]** Sugar syrup with *B. asteroides* DSM 20431; **[Bi]** Sugar syrup with *B. indicum* DSM 20214; **[Ak]** Sugar syrup with *A. kunkeei* LMG 30566; **[Lp]** Sugar syrup with *L. plantarum* LMG 30567; **[Lj]** Sugar syrup and *L. johnsonii* LMG 30568; **[BM]** Sugar syrup with bacterial mixture; **[PBS]** sugar syrup diet and honey bees injected with PBS **[FM]** Fermented media. Asterisks indicate significant differences.

4.3. Results and statistical details on "Assay 2"
4.3.1. Survival

Figure S3

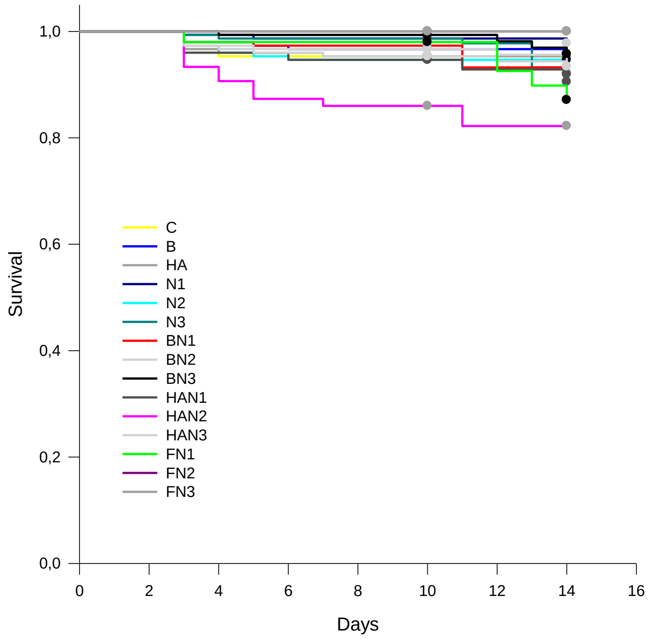

Figure S3. **Survival curves for Assay 2**. [N1] N. ceranae infection with 500 spores; [N2] N. ceranae infection with 5000 spores; [N3] N. ceranae infection with 50000 spores; [B] beneficial bacteria mixture × 500 spores [BN1], × 5000 spores [BN2] or × 50000 spores individually inoculated [BN3]; [HA] Hive Alive® × 500 spores [HAN1], × 5000 spores [HAN2] or × 50000 spores individually inoculated [HAN3]; [F] Fumagillin Salt × 500 spores [FN1], × 5000 spores [FN2] or × 50000 spores individually inoculated [FN3]. There were no statistical differences comparing treatments with the corresponding control under the same infective dose of spores (Kaplan-Meier Survival Analysis: Log-Rank; Statistic: 50.904; DF: 14; p <0.01).

4.3.2. Consumption rate

Table S5

Treatments	Avg.	Desv
**C**	28.56	0.27
**B**	30.99	0.30
**HA**	37.35	0.37
**CN1**	24.83	0.30
**CN2**	26.40	0.23
**CN3**	26.09	0.25
**BN1**	26.67	0.24
**BN2**	24.46	0.30
**BN3**	28.32	0.20
**HAN1**	28.34	0.30
**HAN2**	25.82	0.26
**HAN3**	26.91	0.16
**FN1**	27.31	0.20
**FN2**	26.37	0.33
**FN3**	25.44	0.30

Table S5 Consumption rates for Assay 2. Average of consumption rates for each treatment (mg/bee/day). No statistical differences were found (ANOVA, Statistic: 1.123, DF: 5, p value: 0.39). Overall significance level of p<0.05.

4.3.3. *Nosema ceranae* development

Table S6

	Log$_{10}$ *N. ceranae* spores (Day 9)			Log$_{10}$ *N. ceranae* spores (Day 14)		
**Treatment**	**Average**	**St. Dev**	**Median**	**Average**	**St. Dev**	**Median**
**C**	2.55	0.45	2.52	2.60	1.20	2.82
**B**	1.94	0.20	2.00	0.86	0.84	0.75
**HA**	1.69	0.53	1.69	1.50	1.41	1.59
**N1**	3.74	1.04	4.07	4.75	2.01	3.94
**N2**	7.64	1.95	8.44	5.42	2.59	4.72
**N3**	8.05	1.13	8.41	8.40	1.07	8.72
**BN1**	4.28	1.60	3.88	4.23	0.95	4.19
**BN2**	7.05	1.88	8.24	7.35	1.54	8.14
**BN3**	8.32	1.23	8.70	8.18	1.06	8.52
**HAN1**	5.89	1.94	5.07	6.20	2.04	5.06
**HAN2**	7.27	2.15	8.53	7.09	1.80	7.67
**HAN3**	8.11	1.15	8.44	7.33	1.96	8.37
**FN1**	3.54	0.41	3.54	3.80	0.92	3.96
**FN2**	4.00	0.43	3.95	3.03	0.22	2.93
**FN3**	3.79	0.73	3.65	4.57	0.37	4.63

Table S6: qPCR results on Assay 2. Average, Standard Deviation (St. Dev.) and Median values of *N. ceranae* spores expressed as Log10, on different infection doses. [N1] *N. ceranae* infection with 500 spores; [N2] *N. ceranae* infection with 5000 spores; [N3] *N. ceranae* infection with 50000 spores; [B] beneficial bacteria mixture × 500 [BN1], × 5000 [BN2] or × 50000 spores individually inoculated [BN3]; [HA] Hive Alive® × 500 [HAN1], × 5000 [HAN2] or × 50000 spores individually inoculated [HAN3]; [F] Fumagillin Salt × 500 [FN1], × 5000 [FN2] or × 50000 spores individually inoculated [FN3]. Statistical differences, which are detailed in the manuscript, were found when comparing treatments at the 9th (GLM; DF: 113; p-value: <0.01) and 14th day (Kruskal-Wallis test; χ^2: 108.28; DF: 14; p-value: 2.2×10^{-16}).

Table S7

	Log10 *N. ceranae* spores (Day 9)				Log10 *N. ceranae* spores (Day 14)			
**Treatment**	**Average**	**St. Dev**	**Median**	**%Infected**	**Average**	**St. Dev**	**Median**	**%Infected**
**C**	-	-	-	0.07	1.87	1.81	-	0.07
**B**	-	-	-	0.07	1.87	1.81	-	0.07
**HA**	-	-	-	0.04	1.57	1.81	-	0.04
**N1**	6.63	6.43	-	0.48	6.40	5.68	3.52	0.63
**N2**	7.26	6.64	7.30	0.63	6.97	6.81	6.90	0.96
**N3**	6.94	6.64	6.85	0.78	7.35	7.29	7.00	1.00
**BN1**	6.50	6.35	6.48	0.44	6.98	6.67	6.99	0.48
**BN2**	7.13	6.70	7.12	0.81	7.25	6.67	7.33	0.96
**BN3**	7.30	6.76	7.23	0.78	7.42	7.07	7.30	0.89
**HAN1**	6.55	6.19	-	0.33	6.50	6.15	4.12	0.67
**HAN2**	7.00	6.53	6.92	0.74	7.25	6.61	7.30	1.00
**HAN3**	7.25	6.92	7.19	1.00	7.24	5.84	7.16	1.00
**FN1**	-	-	-	0.00	3.71	3.74	3.52	0.56
**FN2**	2.41	2.82	-	0.22	3.19	3.39	-	0.33
**FN3**	2.65	3.01	-	0.22	2.56	3.04	-	0.11

Table S7. Microscope spore counts on Assay 2. Average, Standard Deviation (St. Dev.) and Median values of *N. ceranae* spores counted under **light microscope**, expressed in Log10, from Assay 1 on different infection doses. Empty cells mean no detection of spores. Labels: [C] Control treatment without spores; [N1] *N. ceranae* infection with 500 spores; [N2] *N. ceranae* infection with 5000 spores; [N3] *N. ceranae* infection with 50000 spores; [B] beneficial bacteria mixture × 500 [BN1], × 5000 [BN2] or × 50000 spores individually inoculated [BN3]; [HA] Hive Alive® × 500 [HAN1], × 5000 [HAN2] or × 50000 spores individually inoculated [HAN3]; [F] Fumagillin Salt, × 500 [FN1], × 5000 [FN2] or × 50000 spores individually inoculated [FN3]. Statistical differences, which are detailed in the manuscript, were found when comparing treatments at the 9th (Kruskal-Wallis test; χ^2: 359.95; DF: 14; p-value: 2.2×10^{-16}) and 14th day (Kruskal-Wallis test; χ^2: 220.94; DF: 14; *p*-value: 2.2×10^{-16}).

4.4. Results and statistical details on "Assay 3"

1.4.1. Survival

Figure S4

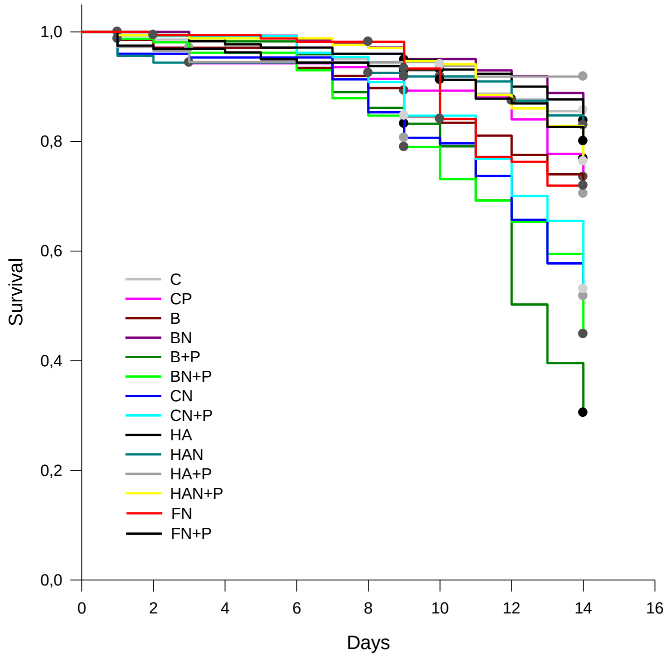

Figure S4. **Survival curves for Assay 3**. [C] Control treatment without infection; [CP] Control treatment without infection, including pollen; [B] beneficial bacteria mixture on syrup; [BN], *N. ceranae* infection with 50,000 spores and a diet of beneficial bacteria mixture [BP] beneficial bacteria mixture on syrup including pollen; [BNP] *N. ceranae* infection with 50,000 spores and a diet of beneficial bacteria mixture on syrup, including pollen; [CN] *N. ceranae* infection with 50,000 spores with no added treatment; [CNP] *N. ceranae* infection with 50,000 spores, including pollen; [HA] Hive Alive®; [HAN] Hive Alive® with *N. ceranae* infection with 50,000 spores; [HANP] Hive Alive® with pollen and *N. ceranae* infection with 50,000 spores; [FN] Fumagillin with *N. ceranae* infection with 50,000 spores; [HANP] Fumagillin with pollen and *N. ceranae* infection with 50,000 spores. There is a statistically significant difference between survival curves (Kaplan-Meier Survival Analysis: Log-Rank; Statistic: 239,625; DF: 13; *p*-value: <0.001).

Table S8

Treatment	Statistic	Unadjusted P Value	Critical Level	Stat. differences?
**C *vs* CP**	0.633	0.426	0.00465	no
**C *vs* CN**	17.241	0.0000329	0.000827	yes
**CP *vs* CNP**	7.578	0.00591	0.00105	no
**CN *vs* CNP**	0.392	0.531	0.00639	no
**B *vs* BP**	10.383	0.00127	0.000967	no
**B *vs* BN**	9.432	0.00213	0.00101	no
**BP *vs* BNP**	2.792	0.0948	0.00165	no
**BN *vs* BNP**	67.119	$2.56E^{-16}$	0.000576	yes
**HA *vs* HAP**	2.592	0.107	0.00177	no

Table S8. **Statistical details for survival curves on Assay 3**. Comparison of survival curves for selected treatments (Log-Rank Test, Statistic= 239,625, DF: 13, *p*-value= 0.001). Pairwise multiple comparison was performed with Holm-Sidak method. Overall significance level of *p*<0.05. For caption details see Fig. S4.

4.4.2. Consumption rate

Table S9

Treatments	Avg.	Desv	Stat. differences versus
**C**	29.70	6.52	
**CP**	27.13	6.97	
**B**	28.00	8.08	
**BN**	27.57	6.72	
**B+P**	23.85	6.77	
**BN+P**	24.98	5.29	
**CN**	23.56	7.01	
**CN+P**	25.58	8.04	
**HA**	23.95	6.04	
**HAN**	22.26	5.91	
**HA+P ***	32.31	10.39	HA+P and HAN
**HAN+P**	22.24	8.07	
**FN ***	16.69	5.99	C, CP, B, BN and HAP
**FN+P ***	15.65	5.42	C, P, B, BN, CNP and HAP

Table S9. **Consumption rates for Assay 3**. Average of consumption rates for each treatment (mg/bee/day). Statistical differences were found (ANOVA, Statistic: DF13; F: 5,387; $p<0.001$). Asterisk indicate treatments with statistical differences vs. the treatments detailed in the last column. All Pairwise Multiple Comparison Procedures (Holm-Sidak method). Overall significance level $p<0.05$. For caption details see Fig. S4.

4.4.3. *Nosema ceranae* development

Table S10

	**Log_{10} *N. ceranae* spore copies (qPCR)**					
	**without pollen feeding**			**pollen feeding (P)**		
**Thesis**	**Average**	**St. Dev**	**Median**	**Average**	**St. Dev**	**Median**
**C**	1.84	0.72	1.84	1.94	0.81	2.03
**B**	2.69	0.79	2.95	2.61	1.08	2.42
**HA**	2.05	0.55	2.00	2.37	0.37	2.52
**CN**	7.50	0.54	7.42	8.33	0.50	8.47
**BN**	7.01	0.49	7.17	7.70	0.22	7.75
**HAN**	6.67	0.51	6.68	7.06	0.11	7.08
**FN**	3.48	0.38	3.54	4.38	0.85	3.99

Table S10 qPCR results for assay 3. Average, Standard Deviation (St. Dev.) and Median values of *N. ceranae* spore's copies expressed in Log_{10}, from Assay 3 (day 9 after infection) on presence and absence of pollen. [C] MRS broth; [P] Pollen; [B] beneficial bacteria mixture; [HA] Hive Alive®; [N] *N. ceranae* infection with 50000 spores; [F] Fumagillin Salt. Statistical differences, which are detailed in the manuscript, were found when comparing between treatments feed with pollen (Kruskal-Wallis test; χ^2: 58.77; DF: 3; *p*-value: 7.96×10^{-11}) or without pollen (Kruskal-Wallis test; χ^2: 108.28; DF: 3; *p*-value: 2.88×10^{-10}).

Table S11

	Log10 (*N. ceranae* spore counts) on microscope					
	without pollen feeding			pollen feeding (P)		
**Thesis**	**Average**	**St. Dev**	**Median**	**Average**	**St. Dev**	**Median**
**C**	0	0	0	0	0	0
**B**	0	0	0	0	0	0
**HA**	0	0	0	0	0	0
**CN**	6.30	6.39	5.99	6.82	6.76	6.70
**BN**	6.62	6.65	6.40	6.98	6.83	6.91
**HAN**	6.12	6.22	5.82	6.62	6.47	6.52
**FN**	2.90	3.60	0	2.87	3.59	0

Table S11. Light microscopy results for assay 3. Average, Standard Deviation (St. Dev.) and Median values of *N. ceranae* spore counts from Assay 3, expressed in Log_{10} for the treatments with positive spore counts or “zero” for the treatments with no detectable spores (day 9 after infection) on the presence or the absence of pollen. [C] MRS broth; [P] Pollen; [B] beneficial bacteria mixture; [HA] Hive Alive®; [N] *N. ceranae* infection with 50000 spores; [F] Fumagillin Salt. Statistical differences, which are detailed in the manuscript, were found when comparing between treatments feed with pollen (Kruskal-Wallis test; χ^2: 54.51; DF: 3; p-value: 8.71×10^{-12}) or without pollen (Kruskal-Wallis test; χ^2: 57.71; DF: 3; *p*-value: 0).

4.5. Results and statistical details on "Assay 4"

4.5.1. Survival

Figure S5

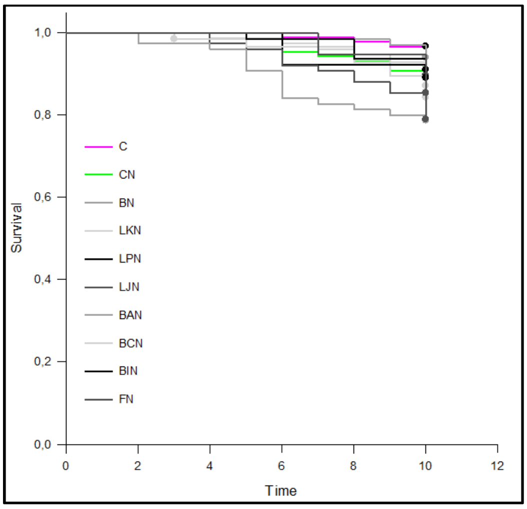

Figure S5. Survival curves for Assay 4. [C] Control treatment without infection; **[CN]** Control treatment with infection; [BN] beneficial bacteria mixture on syrup and *N. ceranae* infection with 50,000 spores; **[LKN]:** Sugar syrup and *A. kunkeei* LMG 30566; **[LPN]:** Sugar syrup and *L. plantarum* LMG 30567; **[LJN]:** Sugar syrup and *L. johnsonii* LMG 30568; **[BAN]:** Sugar syrup and *B. asteroides* DSM 20431; **[BCN]:** Sugar syrup and *B. coryneforme* LMG 30569; **[BIN]:** Sugar syrup and *B. indicum* DSM 20214. **[FN]:** Sugar syrup and antibiotic Fumagillin. Statistical differences between the survival curves were found (Log-Rank Test, Statistic: 19.995, DF: 9, *p*-value: 0.018). No statistical differences were found when comparing treatments versus the infected control **[CN]**. The only statistical difference was found when comparing **C** versus **BAN**. Comparison for survival curves (Log-Rank Test, Statistic= 19.995, DF: 9, *p*-value= 0.018).

4.5.2. Consumption rate

Table S12

Treatments	Avg.	St. Dev	Stat. differences versus
**C**	38.82	6.13	
**CN**	36.13	6.14	
**BN**	38.86	16.55	
**LKN**	43.98	11.93	
**LPN**	34.18	15.19	
**LJN**	53.91	13.90	
**BAN***	55.88	14.91	LP
**BCN***	65.80	23.42	CN, BN, LP
**BIN**	50.14	9.28	
**FN**	46.76	16.69	

Table S12. **Consumption rates for Assay 4**. Average of consumption rates for each treatment (mg/bee/day). Statistical differences were found (ANOVA, Statistic H: 32.287; DF: 8; *p*<0.001). [CN] Sugar syrup; [BN] Sugar syrup and beneficial bacteria mixture; [AKN]: Sugar syrup and *A. kunkeei* LMG 30566; [LPN]: Sugar syrup and *L. plantarum* LMG 30567; [LJN]: Sugar syrup and *L. johnsonii* LMG 30568; [BAN]: Sugar syrup and *B. asteroides* DSM 20431; [BCN]: Sugar syrup and *B. coryneforme* LMG 30569; [BIN]: Sugar syrup and *B. indicum* DSM 20214. [FN]: Sugar syrup and antibiotic Fumagillin. Asterisk indicate treatments with statistical differences vs. the treatments detailed in the last column. All Pairwise Multiple Comparison Procedures (Holm-Sidak method). Overall significance level *p*=0.05.

4.5.3. *Nosema ceranae* development

Table S13

	Log_{10} *N. ceranae* spores (Day 9)		
**Theses**	**Average**	**St. Dev**	**Median**
**C**	3.48	0.78	3.45
**CN**	7.12	0.24	7.09
**BN**	6.49	0.24	6.45
**LKN**	6.13	0.53	6.09
**LPN**	6.77	0.62	6.94
**LJN**	7.10	0.49	6.98
**BAN**	7.56	0.88	7.78
**BCN**	8.38	0.30	8.39
**BIN**	6.83	1.20	7.30
**FN**	5.24	0.21	5.19

Table S13. Results on qPCR from Assay 4. Average, Standard Deviation (St. Dev.) and Median values of *N. ceranae* spores expressed in Log_{10}, on the comparison of control treatment and beneficial bacteria mixture against its single component (Single bacteria strains). [N] *N. ceranae* infection of 50000 spores; [CN] Sugar syrup; [BN] Sugar syrup and beneficial bacteria mixture; [AKN]: Sugar syrup and *A. kunkeei* LMG 30566; [LPN]: Sugar syrup and *L. plantarum* LMG 30567; [LJN]: Sugar syrup and *L. johnsonii* LMG 30568; [BAN]: Sugar syrup and *B. asteroides* DSM 20431; [BCN]: Sugar syrup and *B. coryneforme* LMG 30569; [BIN]: Sugar syrup and *B. indicum* DSM 20214. [FN]: Sugar syrup and antibiotic Fumagillin. Statistical paired comparisons, detailed in the manuscript, were performed by confronting data from control and treated honey bees (GLM Test; DF: 100; p-value 2×10^{-16}).

Table S14

	Log_{10} *N. ceranae* spores (Day 9)		
**Theses**	**Average**	**St. Dev**	**Median**
**C**	0.00	0.00	0.00
**CN**	6.56	6.24	6.47
**BN**	6.45	5.67	6.27
**LKN**	6.36	5.95	5.83
**LPN**	6.29	4.91	5.79
**LJN**	6.56	5.82	6.28
**BAN**	6.55	5.83	6.47
**BCN**	6.39	6.01	5.89
**BIN**	6.45	5.99	6.14
**FN**	3.80	3.82	0.00

Table S14. Results on microscope counts from Assay 4. Average, Standard Deviation (St. Dev.) and Median values of *N. ceranae* spores expressed in Log_{10}, on the comparison of control treatment and beneficial bacteria mixture against its single component (Single bacteria strains). [N] *N. ceranae* infection of 50000 spores; [CN] Sugar syrup; [BN] Sugar syrup and beneficial bacteria mixture; [AKN]: Sugar syrup and *A. kunkeei* LMG 30566; [LPN]: Sugar syrup and *L. plantarum* LMG 30567; [LJN]: Sugar syrup and *L. johnsonii* LMG 30568; [BAN]: Sugar syrup and *B. asteroides* DSM 20431; [BCN]: Sugar syrup and *B. coryneforme* LMG 30569; [BIN]: Sugar syrup and *B. indicum* DSM 20214. [FN]: Sugar syrup and antibiotic Fumagillin. Statistical paired comparisons, detailed in the manuscript, were performed by confronting data from control and treated honey bees (Kruskal-Wallis Test; χ^2: 25.727; DF: 8; p-value: 0.001).

4.6. Results and statistical details on "Assay 5"

4.6.1. Worker bees recovered from in-field assay

Table S15

	Artificially infected honey bees					Naturally infected honey bees				
**Treat.**	**num. recovered**	**% Infection**	**Average**	**St. Dev.**	**Median**	**num. recovered**	**% Infection**	**Average**	**St. Dev.**	**Median**
**C**	40	98	7.59	7.32	7.58	76	88	7.34	6.35	7.14
**B**	47	90	7.37	7.03	7.23	29	77	7.16	7.03	7.20
**BHA**	15	100	7.54	7.29	7.45	52	75	7.27	7.04	7.14
**HA**	0					56	66	7.15	6.93	6.37

Table S15. Results on microscope counts from Assay 5. Average, Standard Deviation (St. Dev.) and Median values of *N. ceranae* spores expressed in Log_{10}, from a semi-field assay, at 18th day. [N] *N. ceranae* infection of 50,000 spores; The sprayed treatments consisted in: [C] Sugar syrup; [B] Sugar syrup and beneficial bacteria mixture; [HA]: Sugar syrup and Hive Alive®; [BHA]: Sugar syrup, beneficial bacteria mixture and Hive Alive®.

Table S16

	Artificially infected honey bees				Naturally infected honey bees			
**Sampling date/analysis**	**Statistic Test**	χ^2	**Degree of freedom**	**Intercept p-value**	**Statistic Test**	χ^2	**Degree of freedom**	**Intercept p-value**
**Day 9 / qPCR**	GLM	N.A.	106	2×10^{-16}	GLM	N.A.	97	2×10^{-16}
**Day 18 / qPCR**	GLM	N.A.	96	2×10^{-16}	GLM	N.A.	95	2×10^{-16}
**Day 18 / microscope**	GLM	N.A.	63	2×10^{-16}	GLM	N.A.	141	6.97×10^{-12}

Table S16. Details on statistical analysis for Assay 5. Statistical differences, which are detailed in the manuscript, were found when comparing treatments at the 9th (Kruskal-Wallis test; χ^2: 359.95; DF: 14; p-value: 2.2×10^{-16}) and 18th day (Kruskal-Wallis test; χ^2: 220.94; DF: 14; *p*-value: 2.2×10^{-16})

Reference:

[83] Huang WF, Solter LF (2013) Comparative development and tissue tropism of *Nosema apis* and *Nosema ceranae*, J Invertebr Pathol 113(1):35–41
